# Biocompatible PANI-Encapsulated Chemically Modified Nano-TiO_2_ Particles for Visible-Light Photocatalytic Applications

**DOI:** 10.3390/nano14070642

**Published:** 2024-04-07

**Authors:** Nefeli Papadopoulou-Fermeli, Nefeli Lagopati, Maria-Anna Gatou, Evangelia A. Pavlatou

**Affiliations:** 1 Laboratory of General Chemistry, School of Chemical Engineering, National Technical University of Athens, Zografou Campus, 15772 Athens, Greecemgatou2@mail.ntua.gr (M.-A.G.); 2 Laboratory of Biology, Department of Basic Medical Sciences, Medical School, National and Kapodistrian University of Athens, 11527 Athens, Greece; 3 Biomedical Research Foundation, Academy of Athens, 11527 Athens, Greece

**Keywords:** PANI/N-TiO_2_, PANI/Ag-TiO_2_, composite materials, functional biomaterials, conducting polymers, semiconductors, photocatalytic activity, visible light, biocompatibility

## Abstract

Polyaniline (PANI) constitutes a very propitious conductive polymer utilized in several biomedical, as well as environmental applications, including tissue engineering, catalysis, and photocatalysis, due to its unique properties. In this study, nano-PANI/N-TiO_2_ and nano-PANI/Ag-TiO_2_ photocatalytic composites were fabricated via aniline’s oxidative polymerization, while the Ag-and N-chemically modified TiO_2_ nanopowders were synthesized through the sol–gel approach. All produced materials were fully characterized. Through micro-Raman and FT-IR analysis, the co-existence of PANI and chemically modified TiO_2_ particles was confirmed, while via XRD analysis the composites’ average crystallite size was determined as ≈20 nm. The semi-crystal structure of polyaniline exhibits higher photocatalytic efficiency compared to that of other less crystalline forms. The spherical-shaped developed materials are innovative, stable (zeta potential in the range from −26 to −37 mV), and cost-effective, characterized by enhanced photocatalytic efficiency under visible light (energy band gaps ≈ 2 eV), and synthesized with relatively simple methods, with the possibility of recycling and reusing them in potential future applications in industry, in wastewater treatment as well as in biomedicine. Thus, the PANI-encapsulated Ag and N chemically modified TiO_2_ nanocomposites exhibit high degradation efficiency towards Rhodamine B dye upon visible-light irradiation, presenting simultaneously high biocompatibility in different normal cell lines.

## 1. Introduction

Industrialization’s rise in both developing and developed nations has posed a significant challenge in the form of environmental pollution. While the industrial revolution has brought advancements in improving quality of life, its detrimental impact on the environment cannot be overlooked [1,2]. Several diseases and disorders, such as cancer and neurodegenerative diseases, are still handled with conventional approaches that inevitably lead to undesirable side effects [1]. Thus, alternative solutions are proposed in order to answer some aspects of these main scientific problems. Therefore, in this framework, a considerable number of inorganic semiconductors have been developed to be used in environmental and recently in biomedical applications.

Organic dyes employed across various sectors, including paper, textile, pharmaceuticals, food, and plastic industries, constitute a significant contributor to water contamination due to their resistance to biodegradation and considerable toxicity, posing risks to both human health and aquatic ecosystems [3]. Dyes’ decomposition proves challenging owing to their resilience against heat, irradiation, as well as oxidizing agents [4]. In order to remove the aforementioned contaminants from wastewater, thus facilitating wastewater reuse, which will provide important financial advantages through decreased expenses related to wastewater disposal and irrigation, various treatment technologies have been explored [2,5]. Nevertheless, numerous of these methods result in the production of unwanted disinfection byproducts, with certain types being labeled as potential carcinogens. Advanced oxidation processes have emerged as a novel technology viable for sewage remediation and disinfection [6]. Among them, heterogeneous catalysis has received great attention in antipollution technology and in biomedicine, utilizing inorganic semiconductors [1,2,5]. Most of them are oxides, and their performance relies on the engineering of their electronic structure and heterojunction formation, which modify the surface properties of the noble metals.

Photocatalysis is characterized as the fusion of photochemistry and catalysis. This scientific domain gained prominence when Fujishima and Honda conducted experiments involving the photolysis of H_2_O into sustainable fuels (H_2_ and O_2_) utilizing a TiO_2_ electrode within an electrochemical setup. Ever since, the application of heterogeneous photocatalysis using TiO_2_ has been extensively acknowledged and utilized as an effective approach for eradicating bacteria and breaking down both organic and inorganic pollutants [7], since TiO_2_ exhibits certain characteristics, such as photostability, enhanced specific surface area, non-toxicity, and cost efficiency [5]. Nonetheless, due to some known limitations, such as enhanced electron (e^−^)–hole (h^+^) pair recombination rate, as well as decreased visible-light sensitivity, various types of alterations have been applied for elevating TiO_2_’s photocatalytic efficiency. Photocatalysts responsive to visible light offer an effective solution for addressing environmental challenges and some health issues based on photodynamic therapy by harvesting energy from visible light. Thus, the emergency of TiO_2_’s chemical alteration is based on utilizing a broader spectrum of solar irradiation, encompassing visible light as well [8,9,10,11,12,13,14]. In fact, visible light makes up 44% of sunlight’s composition. For the exploitation of solar energy, photocatalysts’ development possessing a narrow band gap and an extensive light absorption range, as well as being photostable, chemically inert, reusable, and cost-efficient, is therefore of great importance [15]. Doping with metals and non-metals lies among the most commonly used approaches to enhancing TiO_2_’s activity, when exposed to visible light [16,17].

Among several recently developed photocatalysts, it is noteworthy to mention the conjugated polymer nanostructures (CPNs) with fascinating backbones, consisting of alternating single and double conjugated bonds [18]. CPNs have garnered widespread interest as resilient, metal-free materials responsive to visible light, particularly in the realms of environmental mitigation and solar energy conversion [18]. Nevertheless, conducting polymers typically exhibit solid-state characteristics and are typically non-soluble in conventional solvents. Consequently, conventional methods, such as blending, mixing in solution, and melting, pose challenges for the fabrication of conductive polymer/inorganic nanocomposites [19]. An alternative approach involves encapsulating inorganic nanoparticles within the shell of conductive polymers, offering a viable method for fabricating conductive polymer/inorganic particle nanocomposites [20]. This technique has been employed to encapsulate various metals and metal oxides, resulting in a range of nanocomposites. By carefully designating polymers’, as well as inorganic nanoparticles’ types, the physicochemical attributes of these composites can be properly adjusted.

Advanced photocatalysts demonstrate improved photocatalytic performance due to their distinct structures and compositions. Many notable photocatalysts have been specifically engineered to respond to visible light, enabling the use of solar energy for environmental cleansing regarding antibiotics and several pollutants. By blending different semiconductor materials with complementary characteristics, synergistic outcomes can be attained, leading to enhanced separation of charges, greater surface area, and enhanced catalytic efficacy. This strategy has played a pivotal role in propelling advancements in the realm of photocatalysis for environmental remediation [21,22].

Typically, the epoch of intrinsically conductive polymers commenced upon the discovery of polyacetylene in 1958 [23]. Recent studies revealed that conductive polymers, such as polyaniline (PANI), can remarkably improve TiO_2_’s photosensitivity of under visible-light excitation. Composites of PANI and nano-TiO_2_ can integrate some ideal properties of PANI, as well as nano-TiO_2_ particles, with prospective applications in coatings possessing conductivity, charge storage, electrocatalysis, electrochromic devices, as well as photovoltaic cells. Conductive polymers characterized by extended π-conjugated electron systems indicate absorption within the visible-light spectrum, being able to act as efficient photosensitizers for TiO_2_ [16,17,24]. Hence, by integrating TiO_2_ semiconductors with conductive polymers, enhanced photocatalytic effectiveness can be achieved upon visible-light exposure. This enhancement occurs because conductive polymers improve sunlight’s utilization within the visible-light spectrum, as well as reduce TiO_2_’s e^−^-h^+^ recombination rate, prompting a more effective elimination of organic pollutants from the environment [8] and generally better photocatalytic performance. PANI constitutes a commonly chosen conductive polymer to be combined with TiO_2_, given its environmental robustness, facile synthesis, non-toxicity, cost-efficiency, adjustable doping/de-doping chemistry, as well as advanced physicochemical properties [16,17].

PANI was introduced into usage during the mid-20th century. In an article by Brown and colleagues (1947) discussing aniline’s (in the vapor phase) catalytic oxidation, they referenced “polyaniline products such as aniline black” [23]. It is considered among the most thoroughly investigated conductive polymers because, among the aforementioned properties, it also possesses adjustable and readily reversible electrical attributes and redox-active moieties and allows a diversity of structural forms. Actually, PANI is both an intrinsically conducting polymer and an organic semiconductor. The molecular structure of polyaniline typically contains benzenoid, quinonoid, or both at distinct proportions [24]. It appears in different states depending on the oxidation level, with PANI emeraldine salt (PANI-ES) being identified as the most robust and conductive form [23,25]. Additionally, PANI hydrochloride and PANI base have demonstrated notable biocompatibility regarding dermal irritation and sensitization [26].

The applications of PANI are various, including potential use in environmental remediation by dye degradation and other such as chemical (bio)sensors, optical displays (e.g., non-linear optical and light-emitting devices, radar absorbing materials), energy and memory devices (e.g., chargeable batteries, shielding of electromagnetic interference, erasable optical information storage, digital memory devices), catalysts, solar cells, coatings (e.g., asymmetric film membranes, antistatic, and anticorrosion coatings), electrochromic devices (e.g., electromechanical actuators, electrorheological—fluids), organic light-emitting diodes, Schottky diodes, transistors, and supercapacitors [27,28]. Recently, electrically conductive polymeric materials, and particularly PANI, have additionally attracted significant interest among researchers towards investigating their prospective applications in biomedicine (biosensors, drug delivery, tissue engineering, etc.) [29].

However, the photocatalytic efficiency of PANI has not yet been examined thoroughly. Thus, the research about PANI would open new possibilities for this material to be utilized in photocatalysis [30,31]. In the present study, nano-N-TiO_2_ and nano-Ag-TiO_2_ powders were prepared applying a sol–gel technique. PANI-encapsulated chemically modified TiO_2_ nanocomposites were synthesized through aniline’s in situ chemical oxidation via utilizing ammonium persulfate as an oxidative agent. To achieve the synergistic effect of PANI-TiO_2_, the conductive state of the polymer is required, as is the ideal size of the aggregates. Nevertheless, PANI’s conductivity, as well as the sizes of its aggregates are influenced by the conditions during synthesis. The synthesized nanocomposites were fully characterized and investigated regarding their ability to photocatalytically degrade rhodamine B under visible-light irradiation, while they were also tested regarding their toxicity in different normal cell lines. The ultimate scope of this study is to develop biocompatible materials that could be used either in environmental or biomedical applications exhibiting photocatalytic behavior under visible-light irradiation, avoiding any harmful effects in health tissues, in water, and in any living organism. An application that could utilize these materials is within the field of photocatalytic paints, which pose significant constraints on the integration of photocatalysts, due to the strong limitations that are encountered during their development.

## 2. Materials and Methods

### 2.1. Materials’ Synthesis and Preparation

#### 2.1.1. Synthesis of Ag-TiO_2_ and N-TiO_2_ Inorganic Particles

For synthesizing both Ag-TiO_2_ and N-TiO_2_ powders, an experimental path was followed utilizing the sol–gel method, according to the protocols that have been described in detail in our recent articles [32,33].

#### 2.1.2. Synthesis of PANI/ N-TiO_2_ and PANI/Ag-TiO_2_ Composites

**Figure 1:**
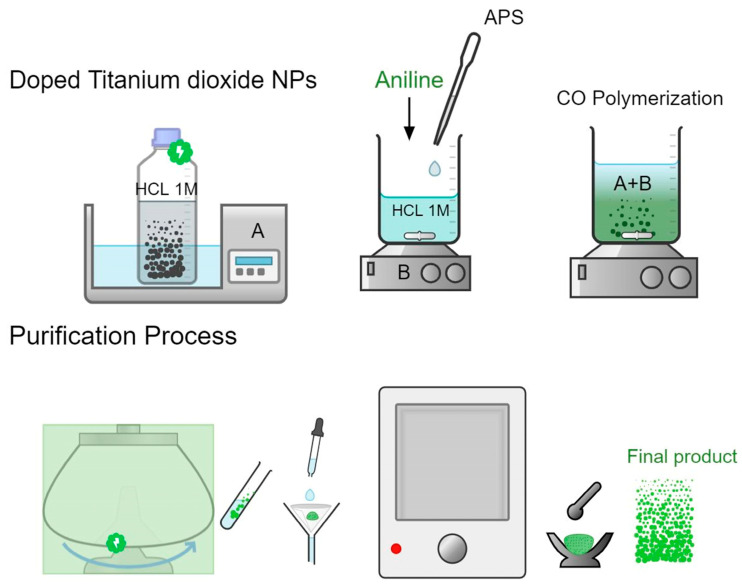
Diagrammatic depiction of the synthetic process for PANI/N-TiO_2_ and PANI/Ag-TiO_2_ composite materials.

PANI/N-TiO_2_ and PANI/Ag-TiO_2_ composites were synthesized according to the in situ chemical oxidative polymerization approach. A proper amount (0.8 g) of the produced inorganic particles (N-TiO_2_ or Ag-TiO_2_) was dispersed into a HCl (1 M) solution (200 mL), containing 0.003 mol of aniline (C_6_H_5_NH_2_, 99.5% Penta Chemicals, Czech Republic), applying ultrasonic vibrations for 2 h, followed by vigorous stirring for one hour. Ammonium persulfate (APS) (0.003 mol) ((NH_4_)_2_S_2_O_8_, ≥98.0%, Penta Chemicals, Czech Republic) was already dissolved in a HCl (1 M) solution (50 mL) and was then poured into the previous dispersion dropwise, under stirring. A few minutes later, the solution’s color became pale blue, unveiling PANI’s synthesis via an oxidation reaction. Ultimately, the solution obtained a greenish color. The polymerization was permitted to continue at 25 °C for 24 h. Subsequently, the resultant reaction mixture underwent filtration and was rinsed thrice with distilled water. Afterwards, the acquired products were subjected to vacuum drying (60 °C, 24 h), yielding a fine powder (Figure 1) [34].

**Figure 2:**
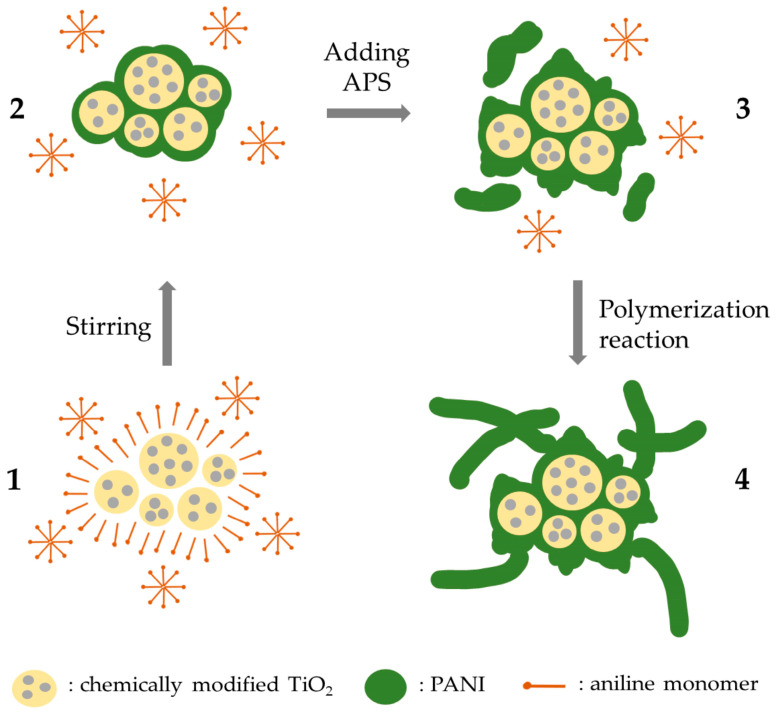
Diagrammatic illustration of PANI/chemically modified TiO_2_ composites’ synthetic route.

The entire composite nanoparticles’ synthetic process involved two distinct stages. Initially, PANI’s polymerization occurred exclusively superficially onto chemically modified TiO_2_ particles, facilitated by the oxidation sites present on the particles’ surface (Figure 2). It is noteworthy that PANI’s synthesis did not occur within the bulk HCl solution. This initial phase also facilitated the development of PANI/TiO_2_ composites imitating a core (TiO_2_)/shell (PANI)-like structure. In the subsequent stage, by introducing ammonium persulfate as an oxidizer towards aniline monomers, a thicker layer of PANI is formed on the surface of TiO_2_, as well as some aniline oligomers within the bulk HCl solution. The remaining oligomers and monomers that were assembled on the bulk also attach onto the formed layer on TiO_2_’s surface, and they start to grow up more and more, participating in the polymerization reaction [34,35].

#### 2.1.3. Preparation of Rhodamine B (RhB) Solution

For assessing the photocatalytic efficacy of the as-synthesized nanocomposites, rhodamine B served as the representative pollutant for examining photocatalytic degradation upon visible-light exposure. Particularly, RhB (C_28_H_31_CIN_2_O_3_, Penta-Chemicals Unlimited, Prague, Czech Republic) solution (1 L) was prepared using distilled water [32]. RhB has been proven to be very resistant towards TiO_2_ compared to other pollutants, such as Brilliant Green and Methylene Blue, indicating that it is stable and thus suitable to be used in photocatalytic trials [36].

### 2.2. Characterization Techniques

#### 2.2.1. Instrumentals

The XRD analysis (Bruker D8 Advance, Bruker, Germany) was performed utilizing the following conditions during measurements: 2θ angle = 5–100°, scanning rate = 0.1° per minute, λ_Cu-Kα_ = 1.5418 Å, voltage = 40 kV, as well as current = 40 mA. In addition, measurements under the same experimental conditions were conducted using an NIST standard LaB_6_ powder (Malvern Panalytical Ltd., Malvern, UK) as the reference material for calculating the instrumental broadening considered for the Scherrer equation calculations.

Raman spectroscopy was fulfilled by deploying a micro-Raman apparatus (inVia, Renishaw, Wotton-under-Edge, Gloucester, UK) possessing two excitation sources (solid-state lasers) functioning at wavelengths equal to 532 nm and 633 nm, respectively, and with an average power of ≈50 mW. Raman analysis was conducted at ambient temperature, and a x50 short distance magnification lens was employed to direct the laser beam onto each examined sample’s surface. Reduced excitation power (10%) was exploited to minimize surficial temperature increase caused by the laser. Frequency adjustments were standardized by an internal reference material (Si). For each sample, 2–3 spots were measured. The exposure time was set to 30 s, with an average of 3 accumulations, and the recorded spectral range was 100–1800 cm^−1^.

For the FTIR analysis (Brucker alpha II, Platinum-ATR, Brucker, Berlin, Germany), a minimum of 16 scans were employed to acquire each spectrum. FTIR spectra were documented within the range 250–3750 cm^−1^ (spectral resolution: 4 cm^−1^).

The bandgap values of the as-synthesized particles were estimated utilizing a UV-Vis spectrometer (Jasco UV/Vis/NIR model name V-770, Interlab, Athens, Greece) possessing an integrating sphere, hence facilitating diffuse reflectance measurements.

Zeta potential values were ascertained via dynamic light scattering (Malvern Zetasizer Nano ZS, Malvern Panalytical Ltd., Malvern, UK). A 633 nm laser constituted the incident light, while a 173° scattering angle was employed to record the corresponding intensity.

The surface analysis measurements were carried out in an ultra-high vacuum chamber (P~5 × 10^−10^ mbar) equipped with a SPECS Phoibos 100 hemispherical electron analyzer with a delay line detector (DLD) and an unmonochromised dual-anode Mg/Al X-ray source. The XP spectra were obtained with MgKa (hν = 1253.6 eV), and an analyzer pass energy of 10 eV giving a Full Width at Half Maximum (FWHM) of 0.85 eV for the Ag3d line was used. The analyzed area was a spot of 3 mm diameter, while for spectra collection and treatment, including fitting (a fitting routine was used resulting in the decomposition of each detailed spectrum into individual mixed Gaussian–Lorentzian peaks after a Shirley background subtraction), the commercial software SpecsLab Prodigy (by Specs GmbH, Berlin, Germany) was used.

Finally, transmission electron microscopy (FEI Talos F200i, ThermoFisher Scientific Inc., Waltham, MA, USA) operating at a voltage equal to 200 kV and possessing a windowless EDS microanalyzer (6T/100 Bruker, Hamburg, Germany) was utilized for examining the composite particles’ morphology [32].

#### 2.2.2. Photocatalytic Trials

The photocatalytic effectiveness of the obtained powders and composites upon visible-light exposure was assessed by employing 0.005 g of each product in RhB aqueous solution (0.01 M, 5 mL) at ambient temperature and pH = 7.50 ± 0.01, under constant stirring. Before each photocatalytic trial, pure oxygen (99.999%) was vented via RhB’s solution for 2 h in the absence of light to ensure saturation. The photoreactor utilized featured four parallel lamps positioned 10 cm above each sample’s surface. These lamps were 15 W daylight lamps (350–750 nm, 3 mW/cm^2^, OSRAM GmbH, Munich, Germany). The as-synthesized samples’ absorbance was quantified utilizing a Hitachi U-2001 Spectrophotometer (Hitachi, Tokyo, Japan). The assessment involved determining the fraction of the assessed absorption (A) at each specific time point to the initial absorption (A_initial_) after the nanoparticles had been removed by centrifugation, thereby enabling the concentration fraction’s (C/C_0_) calculation, where C represents RhB’s concentration after a specific duration of photocatalytic trials, and C_0_ corresponds to RhB’s initial concentration measured at λ = 554 nm [32].

### 2.3. Biocombatibility Test

#### 2.3.1. Cell Cultures

Two normal cell lines, HEK293 (human epithelial kidney embryonic cells, LGC Standards GmbH, Wesel, Germany) and FF95 (human foreskin derma fibroblast cell strains, LGC Standards GmbH, Wesel, Germany), were cultured in Dulbecco’s modified Eagle’s medium (DMEM) (Gibco BRL, ThermoScientific, Paisley, UK). The media were augmented with 10%, 1%, and 1% of FBS (fetal bovine serum), L-glutamine, and sodium pyruvate, respectively, as well as antibiotics (Gibco, Paisley, UK). Cells’ incubation was performed at certain conditions (T: 37 °C, humidity: 99%, CO_2_: 5%). In the case of cells’ trypsinization, a trypsin–EDTA mixture (0.05/0.02% *w*/*v*, Gibco, BRL, Life Technologies, ThermoScientific, Paisley, UK) was employed [37].

#### 2.3.2. Cytotoxicity Test

Normal cells’ viability was assessed applying the MTT colorimetric assay (Thiazolyl Blue Tetrazolium Bromide M5655, Sigma-Aldrich, Darmstadt, Germany), as previously depicted [37]. Untreated cells (cells cultured in the medium) were considered a negative control. Also, visible-light photoactivated cells (30 min), in the absence of the examined materials, were utilized as an additional negative control to examine the possible thermal effect caused by the irradiation. Each experimental trial was repeated thrice in triplicate. The non-parametric Kruskal–Wallis test was employed to statistically analyze the obtained results, acknowledging statistical significance for *p* values below 0.05 [38].

## 3. Results and Discussion

### 3.1. Nanocomposites’ Characterization

#### 3.1.1. XRD Analysis

XRD was utilized for estimating the structural attributes, as well as crystallinity, of the as-prepared PANI/chemically modified TiO_2_ samples. In this study, PANI-ES (emeraldine salt) possessing crystallinity was synthesized, which will be called hereinafter PANI_HCL for convenience purposes.

**Figure 3:**
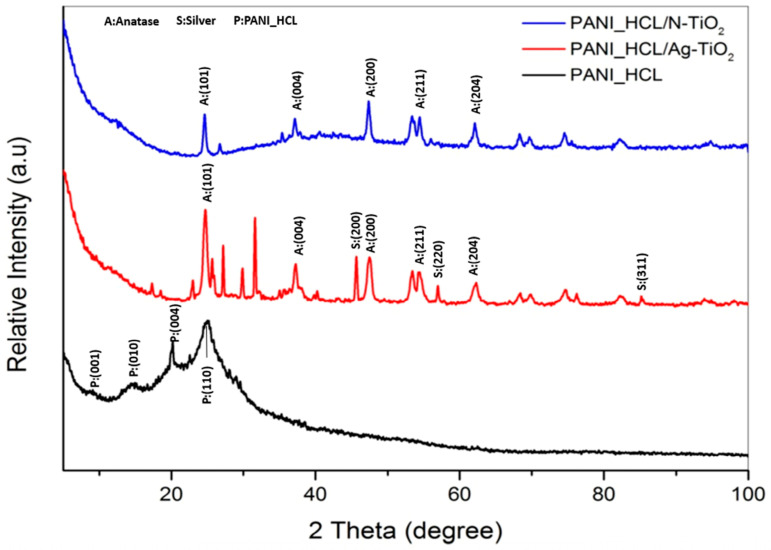
XRD diffractograms of PANI_HCL (black), PANI_HCL/Ag-TiO_2_ (red) and PANI_HCL/N-TiO_2_ (blue).

In the XRD diffraction diagram of PANI_HCL (Figure 3), the peaks present at 2θ = 8.7°, 14.5°, 20°, and 25.2° are attributed to the (001), (010), (100), and (110) crystal planes [17,39], respectively, indicating that pure PANI_HCL presents a semi-crystal structure in accordance with PDF No 03-065-5714.

Regarding PANI_HCL/Ag-TiO_2_ and PANI_HCL/N-TiO_2_ composite samples (Figure 3), the dominant peak spotted at 2θ ≈ 24.7° is assigned to the (101) crystal plane of anatase-TiO_2_, in accordance with PDF No 03-065-5714. In addition, the peaks located at 2θ = ~37.3°, 47.5°, 54,4°, 62.1° are assigned to the (004), (200), (211), and (204) crystal planes of anatase-TiO_2_, respectively (PDF No 03-065-5714). As for the PANI/Ag-TiO_2_ composite sample, the peak observed at 2θ = 45.5°, indicates the (200) plane of metallic silver (Ag^0^), whereas the peaks present at 2θ = 56.9° and 85.2° stand for the (220) and (311) planes of AgCl, respectively [32,40]. Concerning the strength of the peak of the silver phase at 2θ=~38° (111), its intensity could be notably affected by the amorphous section of the polymeric matrix. Furthermore, another peak at approximately 2θ = 37.3° from anatase-TiO_2_ might coincide with the possibly shifted peak linked to the (111) phase of silver [17,41]. The distinctive peaks of pure PANI are also present in the composite samples, thus confirming the successful synthesis of the composite samples. Additionally, comparing the XRD spectra of PANI_HCl/Ag-TiO_2_ and PANI_HCl/N-TiO_2_, the peaks appearing in the PANI_HCl/Ag-TiO_2_ spectrum at 2θ = ~21–33° correspond to interactions between PANI and Ag-TiO_2_, while in the characteristic spectrum of PANI_HCl/N-TiO_2_, they are not as intense, implying a higher percentage of amorphous regions in the PANI_HCl/N-TiO_2_ sample, which is consistent with the transmission microscopy images. This element confirms the different crystallinity percentages. In the case of PANI_HCL/N-TiO_2_ and PANI_HCL/Ag-TiO_2_, the difference in CI values can be attributed to the presence of silver nanoparticles in the Ag-TiO_2_ catalyst. Silver nanoparticles have been shown to promote the crystallization of polyaniline (PANI) during the electrochemical polymerization process. This is because silver nanoparticles can act as nucleation sites for the growth of PANI crystals, leading to a more ordered and crystalline structure. In contrast, the N-TiO_2_ catalyst does not have this effect, resulting in a lower CI value for PANI_HCL/N-TiO_2._

The samples’ average crystallite size was estimated through Scherrer’s equation (Equation (1)):

(1) $$D=\frac{0.89\lambda }{\beta \cos\theta }$$

where D constitutes the average crystallite size, 0.89 stands for Scherrer’s constant, λ constitutes the X-ray wavelength (λ = 1.5418 Å), θ accounts for the diffraction angle, and β corresponds to the full width at half maximum (FWHM), estimated for several well-distinct peaks of anatase-TiO_2_.

Furthermore, crystallinity index (CI %) was evaluated using Equation (2):

(2) $$CI\left(\%\right)=\frac{Area\;of\;all\;crystalline\;peaks}{Area\;of\;all\;crystalline\;and\;amorphous\;peaks}$$

The obtained findings are thoroughly depicted in Table 1.

#### 3.1.2. Micro-Raman Analysis

In polyaniline’s Raman spectrum, there are typically three regions that are prone to both oxidation’s degree and protonation’s level:

1. The region between 1100 and 1210 cm^−1^, where the most prominent vibrations are attributed to C–H bending of benzene or quinone-type rings.
2. The region spanning from 1210 to 1520 cm^−1^, characterized by the presence of characteristic stretching vibrations of C–N, C=N, and C˜N^+^ (˜ symbol implies an intermediate bond amidst a single and a double one).
3. The region between 1520 and 1650 cm^−1^, where the dominant vibrations include C–C and C=C stretching in benzene and quinone-type rings, respectively.

Through spectra observation, it can be verified that, compared to the 532 nm spectrum, where reduced intensities and even the disappearance of bands can be observed, the information obtained from the 633 nm spectrum is more abundant. In compliance with our previous work, the TiO_2_ structure’s typical bands emerge within the 140–617 cm^−1^ range [32,33].

**Figure 4:**
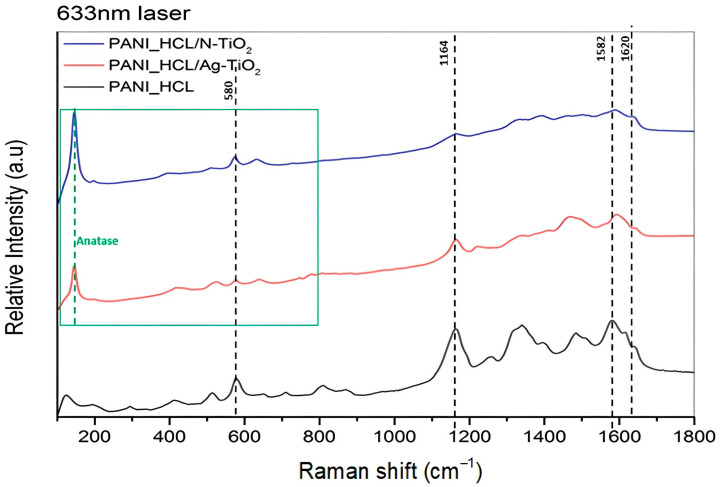
Raman spectra of PANI_HCL (in black), PANI_HCL/Ag-TiO_2_ (in red) and PANI_HCL/N-TiO_2_ (in blue) utilizing a 633 nm laser. The green box is used to indicate the Raman modes of anatase TiO_2_.

As a result, based on the optimal measurement conditions for the as-synthesized samples (excitation wavelength: 633 nm, laser’s intensity: 10%) (Figure 4), the following bands are detected: A band at 1164 cm^−1^ assigned to a quinone ring’s C–H distortion vibration, a band at 1257 cm^−1^ related to a stretching vibration of a quinone structure, and a band located at 1335 cm^−1^ attributed to C~N^+^ stretching vibrations of semi-quinone radical cations in non-localized polaronic structures. Moreover, the band present at ≈1485 cm^−1^ (appearance range within 1480–1468 cm^−1^, where in doped samples the band is shifted to a lower wavenumber) is linked to C=N stretching vibrations of quinoid structures, while the one spotted at 1585 cm^−1^ corresponds to C–C ring stretching vibrations (appearance range of 1591–1593cm^−1^, where in doped samples the band is shifted to a higher wavenumber). Finally, the band present at 812 cm^−1^ is ascribed to the emeraldine structure’s benzene ring distortion vibration. Table 2 summarizes the main bands and their assignment.

The bands observed at 1641, 1556, and 803 cm^−1^, are affiliated with the benzene ring’s deformation in emeraldine salt’s polaronic or bipolaronic form. Moreover, the band located at 815 cm^−1^ signifies the benzene ring’s deformations, while the one observed at 729 cm^−1^ may indicate amine’s deformation, characteristic of emeraldine salt’s bipolaronic form. Also, the observed bands at 518 and 412 cm^−1^ are linked to the ring’s out-of-plane deformations.

**Figure A1:**
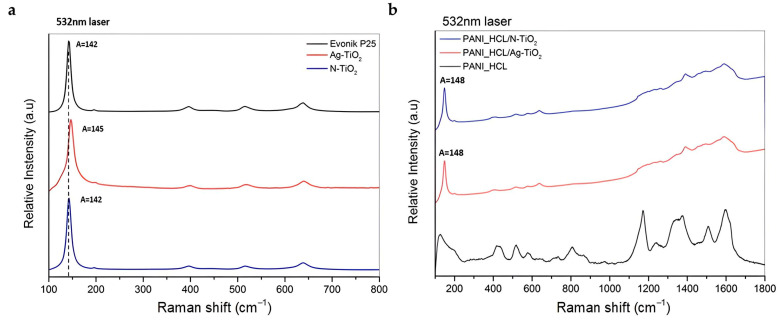
Raman spectra of (**a**) Evonik P25, Ag-TiO_2_ and N-TiO_2_, (**b**) PANI_HCL (in black), PANI_HCL/Ag-TiO_2_ (in red), and PANI_HCL/N-TiO_2_ (in blue) utilizing a 532 nm laser.

The Raman spectrum acquired by utilizing the 532 nm laser line as an excitation source (Figure A1b in Appendix A) is reminiscent of the one obtained using the 633 nm laser line as an excitation source, thus implying that the prevalence of quinonoid units is substantial and not merely a result of resonance enhancement, when using the 633 nm laser line as an excitation source [42]. Nevertheless, it is obvious that more information is captured in the 633 nm laser spectrum (Figure 4) compared to that obtained using the 532 nm laser (Figure A1b in Appendix A). The characteristic bands of TiO_2_ (~140–145 cm^−1^) are shown only in the composite samples with N-TiO_2_ and Ag-TiO_2_, where some peaks are overlapped. In general, the results confirmed that the composites have been successfully synthesized as characteristic bands of polyaniline and TiO_2_ coexist and are compliant with the data acquired through the XRD analysis.

#### 3.1.3. FT-IR Analysis

**Figure 5:**
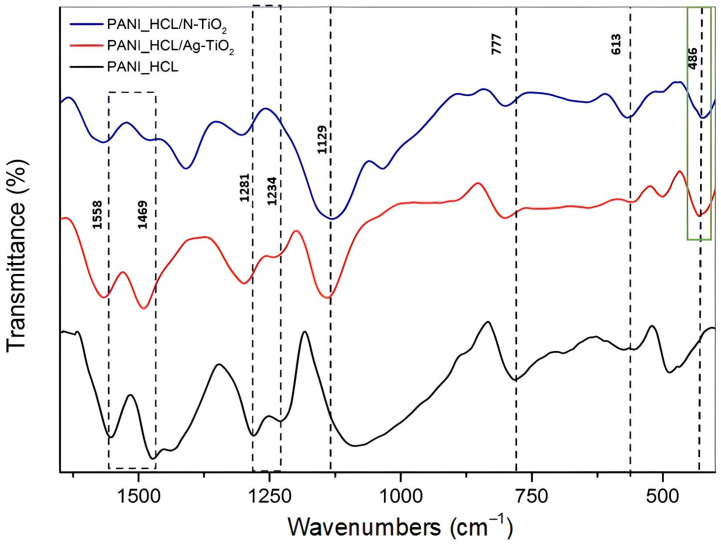
FT-IR spectra of PANI_HCL, PANI_HCL/Ag-TiO_2_ and PANI_HCL/N-TiO_2_.

PANI’s typical band at approximately 3215 cm^−1^, attributed to the N–H stretching vibrations of N-protonated atoms alongside the composites’ PANI chain, appears in all samples, verifying the successful synthesis of the as-mentioned composites (Figure 5) [41].

**Figure 6:**
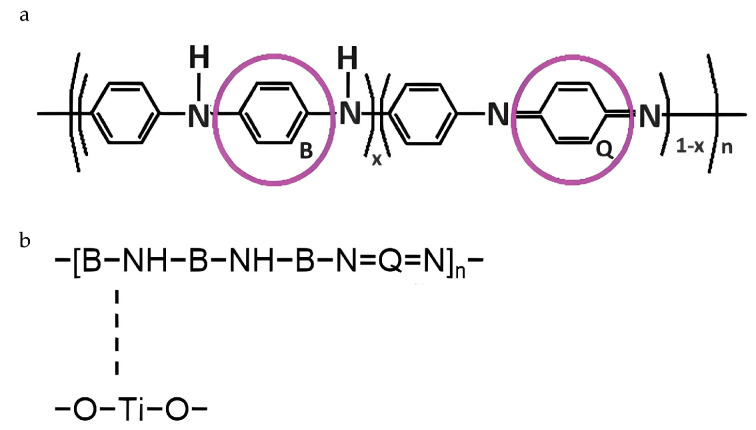
(**a**) PANI’s structural formula, highlighting the quinoid (Q) and benzoid (B) units. (**b**) Established interactions within PANI/TiO_2_ composite among nitrogen (N) in PANI and titanium (Ti) in TiO_2_ (redesigned based on [50]).

In addition, other PANI’s typical bands were spotted at 1558 and 1469 cm^−1^, because of C=C stretching in quinoid (Q) and benzoid (B) units, respectively. The C–N stretching of the benzoid units is responsible for the C–N vibrations in BBB (1235 cm^−1^), as well as for the C–N vibrations in QBB, BQQ, and QBQ (1294 cm^−1^) [43,44], due to secondary amino groups [45]. Furthermore, the band located at 484 cm^−1^ is associated with the aromatic ring’s C–N–C bonding mode [41], while the band spotted at 795 cm^−1^ corresponds to C–H bonds’ vibrations. The bands of pure PANI_HCL sample at 1558 (C=C in Q), 1469, 1281, 777, and 486 cm^−1^ appear shifted to elevated wavenumbers in the composite samples, confirming the interaction among chemically modified TiO_2_ and PANI [45]. The aforementioned observations reveal a correlation between TiO_2_ nanoparticles and polyaniline’s chain, characterized by a π–σ bond interaction. This interaction involves the formation of a σ bond through the intersection of pure PANI’s (PANI_HCL) π molecular orbital with the metal ions’ d-orbital, while a π bond is formed through the intersection of polyaniline’s π* molecular orbital with the metal’s d-orbitals. Additionally, hydrogen bonding among O atoms in TiO_2_ and polyaniline chains within the composites leads to the insertion of TiO_2_ particles into the polymer chain of PANI [45]. Concerning the Ti–O bonds, the bands located at ≈400 cm^−1^ are typical of the O–Ti–O lattice formation [46,47,48,49]. Other characteristic bands are not spotted because of either the strong band intensities corresponding to PANI or the deformation of the crystals due to the successful PANI/chemically modified TiO_2_ synthesis. All examined samples presented notable bands within the 1575–1587 and 1492–1502 cm^−1^ ranges, given the existence of C=N and C=C stretching modes that are characteristic for quinoid and benzoid units (Figure 6). The fraction of intensities between bands associated with C=N and C=C stretching modes suggested that PANI_HCL possessed its conductive state [50]. Table 3 summarizes the main bands and their assignment.

#### 3.1.4. Diffuse Reflectance Spectroscopy (DRS) Analysis

To determine the optical attributes of the as-prepared samples, diffuse reflectance measurements were conducted within the UV–visible range at 25 °C. The band gap energies (E_g_) of the acid-doped PANI-encapsulated chemically modified TiO_2_ samples were estimated utilizing the Kubelka–Munk (Κ-Μ) function [51]. According to the literature, PANI constitutes a characteristic conjugated polymer possessing prolonged π-conjugated e^−^ systems and E_g_ values ranging from 1.8 to 4 eV, depending on its structure, the utilized dopant, as well as the dopant’s concentration. Additionally, PANI constitutes an effective e^−^ donor and h^+^ transporter under visible-light irradiation [39,51,52,53,54,55,56,57,58].

**Figure 7:**
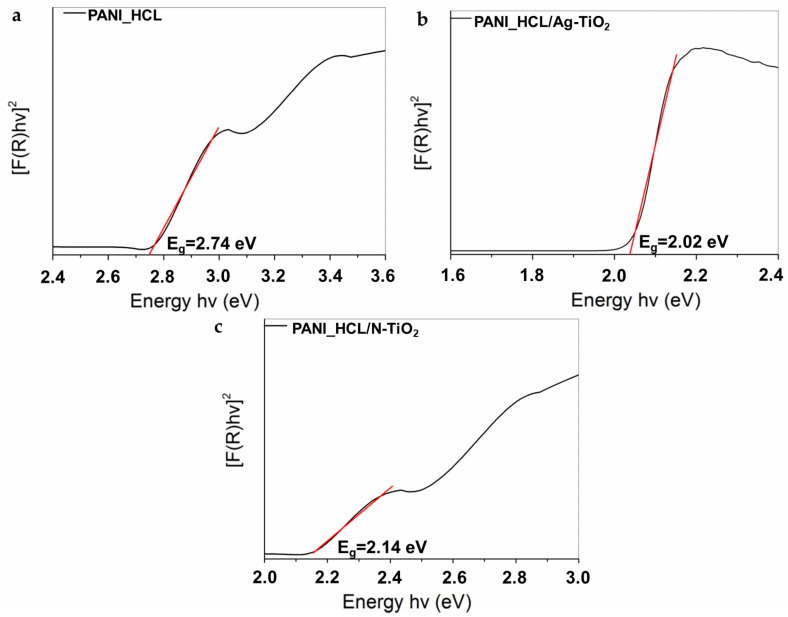
Optical energy band gap (E_g_) of (**a**) PANI_HCL, (**b**) PANI_HCL/Ag-TiO_2_, and (**c**) PANI_HCL/N-TiO_2_ samples.

A precise determination of the E_g_ value is crucial for estimating semiconductors’ photophysicochemical attributes [59]. Thus, a semiconductor’s E_g_ value is assessed through Tauc’s equation (Equation (3)):

(3) $$ahv=A{\left(hv-E_{g}\right)}^{n}$$

where E_g_ constitutes the energy band gap, α represents the absorption’s coefficient, h corresponds to Planck’s constant, v stands for the frequency, and n is equal to 0.5 [33]. E_g_ values were acquired applying the K-M method vs. energy through the extrapolation of the (F(R)hv)^1/2^ vs. hv spectra’s linear region [32,39,59]. In Figure 7, the obtained results are shown, while the measured E_g_ values of the as-synthesized powders are presented in Table 4.

According to the obtained experimental results and the existing literature, the E_g_ value of PANI-based samples is significantly affected by the utilized doping agent. An increase in the interactions between the PANI matrix and the dopants, prompts a simultaneous enhancement in the particle’s conductivity and the charge carriers’ number, while the E_g_ value decreases [51]. However, a decreased E_g_ value corresponds to enhanced solar spectrum utilization [41].

#### 3.1.5. Zeta Potential Measurements

**Figure 8:**
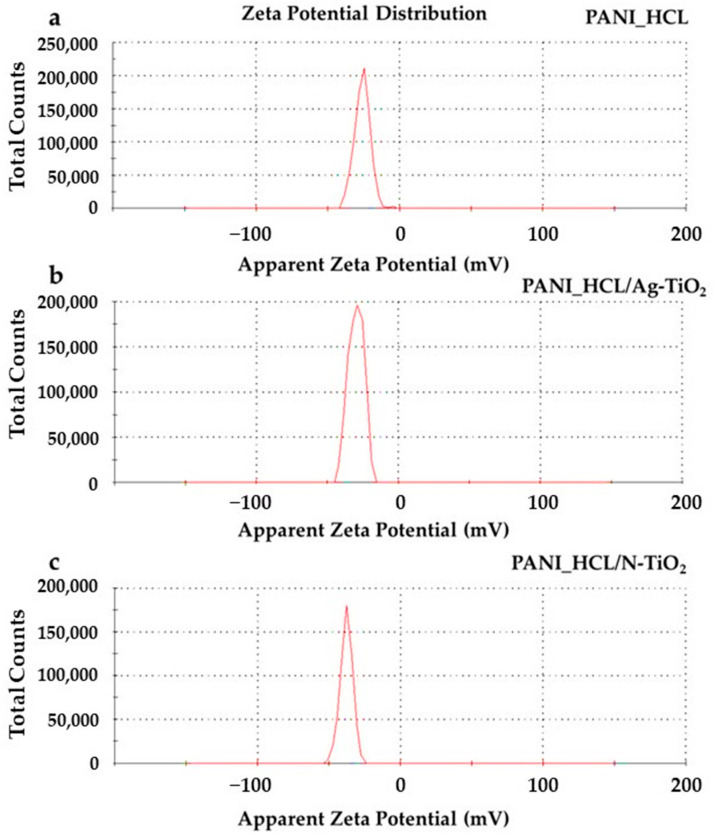
Zeta potential diagrams of the (**a**) PANI_HCL, (**b**) PANI_HCL/Ag-TiO_2_, and (**c**) PANI_HCL/N-TiO_2_ samples.

The measurements for determining the zeta potential were conducted in order to assess the stability of aqueous dispersions under specific pH and temperature conditions. The results revealed samples with relative high stability under these conditions and minimal standard deviation (Table 5 and Figure 8a–c). The aqueous solutions of PANI_HCL-encapsulated Ag or N-doped TiO_2_ nanoparticles exhibited a pH value around 6 at room temperature (Figure 8a–c). In general, high absolute zeta potentials typically indicate good stability, as particles repel each other strongly. The zeta potential of PANΙ-based materials can be affected by several factors, entailing the composite’s composition, the solution’s pH, the existence and type of dopants, as well as the characteristics of the dispersion medium.

#### 3.1.6. XPS Analysis

**Figure 9:**
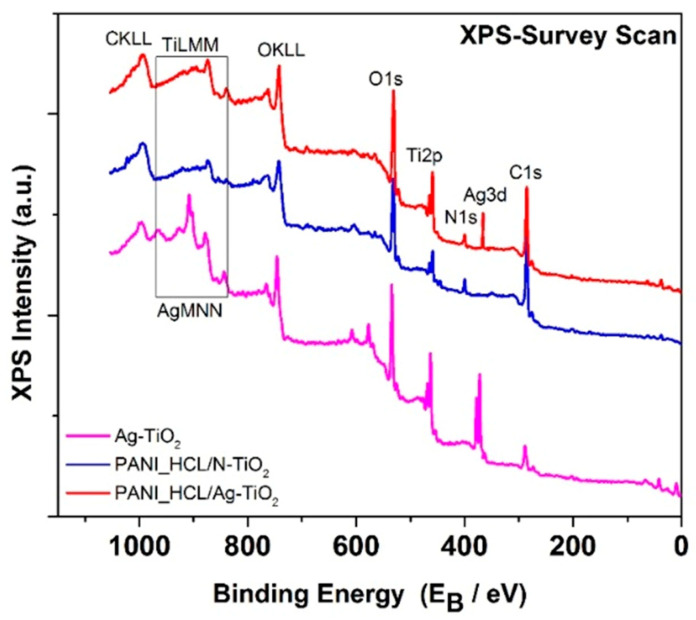
Survey scans from Ag-TiO_2_ (purple line), PANI_HCL/N-TIO_2_ (blue line), and PANI_HCL/Ag-TiO_2_ (red line).

X-ray photo spectroscopy (XPS) was used to examine the composition and chemical states of the elements in the Ag-TiO_2_, PANI_HCL/Ag-TiO_2_, and PANI_HCL/N-TiO_2_ nanocomposites. The X-ray survey scan of the nanocomposites, depicted in Figure 9, revealed the presence of Ag, C, N, Ti, and O elements, as shown by the photoelectron peaks of Ag3d, C1s, N1s, Ti2p, and O1s, respectively.

**Figure 10:**
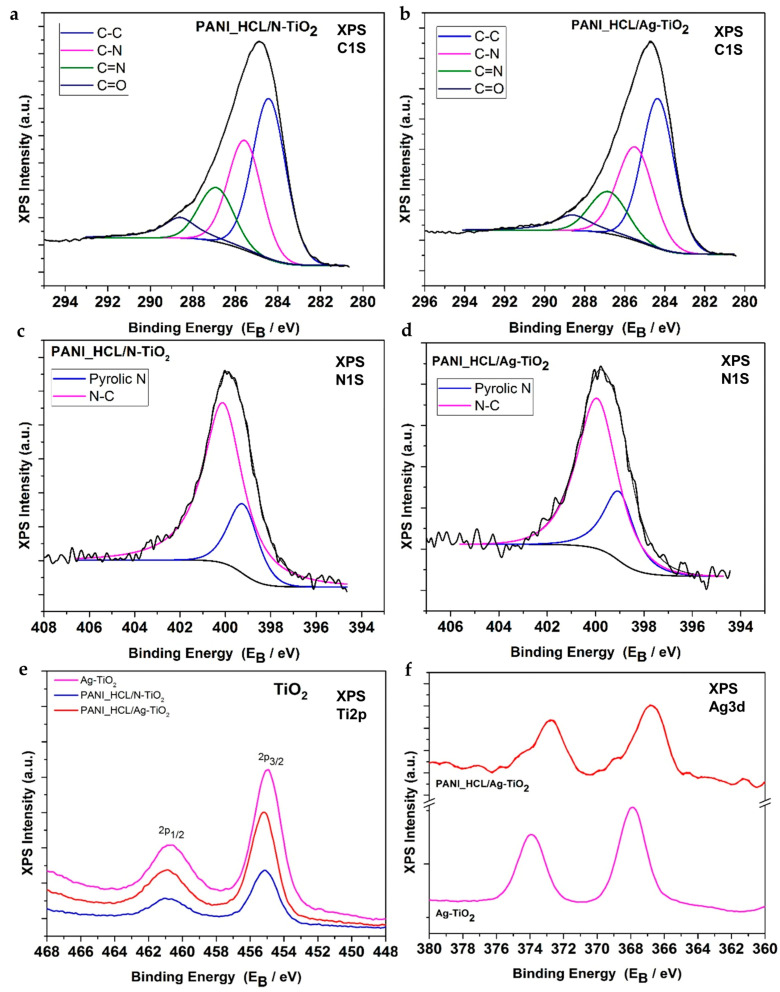
Deconvoluted C1s peak from (**a**) PANI_HCL/N-TIO_2_ and (**b**) PANI_HCL/Ag-TiO_2_ samples. Deconvoluted N1s peak from (**c**) PANI_HCL/N-TIO_2_ and (**d**) PANI_HCL/Ag-TiO_2_ samples. (**e**) Ti2p XPS peak of Ag-TiO_2_ (purple line), PANI_HCL/N-TIO_2_ (blue line) and PANI_HCL/Ag-TiO_2_ (red line). (**f**) Ag3d XPS peak of Ag-TiO_2_ and PANI_HCL/Ag-TiO_2_.

Figure 10a,b show the deconvoluted C1s peak from PANI/N-TIO_2_ and PANI/Ag-TIO_2_ TiO_2_ samples, respectively. In the PANI/N-TIO_2_ and PANI/Ag-TIO_2_ samples, the peak is analyzed into four components corresponding to C–C (284.4 eV), C–N (285.5 eV) bonds in quinoid units, and C–N bonds in benzenoid units, respectively, C=N (286.8 eV), and C=O (288.6 eV) bonds due to PANI [17,41,60]. Moreover, Figure 10c,d shows the deconvoluted N1s peak from PANI_HCL/N-TiO_2_ and Ag PANI_HCL/Ag-TiO_2_ samples, respectively. The peak consists of two components corresponding to pyrrolic nitrogen (399.2 eV) and C–N–C (400.2 eV), which are assigned to PANI [17,41,60]. The latter can also be assigned to N-TiO_2_ bonds [1]. According to our previous study on N-TiO_2_ nanoparticles, the N1s element has been observed at high resolution, revealing information about the chemical state of N–Ti–O bonds. This element can be precisely located at an energy level of 401.07 eV [33].

Figure 10e shows the detailed Ti2p XPS doublet with a spin-orbit splitting (2p3/2–2p1/2) of 5.75 eV and a Ti2p3/2 binding energy centered at 458.8 eV, corresponding to the TiO_2_ chemical state in agreement with our previous work [1]. In the case of Ag-TiO_2_ powder, the Ti2p3/2 and Ti2p1/2 signals were observed at slightly higher binding energies compared to our previous study on N-TiO_2_ nanopowders [33], suggesting a partial transfer of electron density from TiO_2_ to the supported Ag nanoparticles. On the other hand, the Ti2p core levels of PANI_HCL/N-TiO_2_ and PANI_HCL/Ag-TiO_2_ demonstrated a significant shift towards higher binding energy relative to the signals of N-TiO_2_ and Ag-TiO_2_. This shift indicates a strong interfacial adhesion between PANI and the doped TiO_2_ nanoparticles.

The binding energies of the Ag 3d5/2 and Ag 3d3/2 peaks for the Ag-N-TiO_2_ and PANI-Ag-N-TiO_2_ composites provided evidence for the presence of Ag0 (Figure 10f). Collectively, these results demonstrate that the chemical composition of the near-surface region of the composites aligns well with their nominal composition. Moreover, the data support the notion of close contact between PANI and the nanoparticle components (i.e., TiO_2_, N-TiO_2_, and Ag-N-TiO_2_) within the composites. The atomic percentages of Ti, O, C, N, and Ag are calculated from the intensity (peak area) of the XPS peaks, weighted with the corresponding relative sensitivity factors (RSF), taking into account the analyzer’s transmission characteristics, and are shown in Table 6 and Table 7.

#### 3.1.7. TEM Analysis

In the discourse on the composition of polyaniline, delineation occurs across three stages: (a) Nucleation, (b) initial growth, and (c) secondary growth. Traditional approaches yield nanofiber-structured polyaniline due to the linear chain structure, with their formation initiated during nucleation and initial growth phases. These nanofibers subsequently serve as nucleation sites for the additional polymerization of aniline monomers during the secondary growth phase. The outcome of secondary growth often manifests irregularly shaped polyaniline particles. Contrastingly, surface polymerization yields a final product in nanofiber form. During interfacial polymerization, the initial creation of polyaniline nanofibers does not undergo subsequent polymerization. Under mild acidic conditions, oligomeric phenazine-type anilines self-assemble into diverse morphologies, such as flower-like structures or hairy microspheres resembling “rambutan,” while strong acidic conditions yield spherical powders. Moreover, interfacial polymerization engenders various composites by combining polyaniline with other materials such as metal nanoparticles or carbon nanotubes [50,61].

Anionic chlorides adsorb onto the positively charged surface of TiO_2_ nanoparticles, neutralizing their charge. In an acidic milieu (HCl), aniline monomers transform into aniline cations, fostering electrostatic interaction with chloride ions absorbed on TiO_2_ nanoparticles. Polymerization of aniline’s cations culminates in the development of a PANI shell enveloping chemically modified TiO_2_ nanoparticles (@TiO_2_), thereby forming a nanostructured PANI/@TiO_2_ with a core–shell configuration, a phenomenon facilitated through TiO_2_ nanoparticle modification by aniline’s in situ polymerization [62].

TEM delved into the microstructure of PANI/@TiO_2_ composites, revealing the absence of free TiO_2_ nanoparticles regardless of the @TiO_2_ solution’s volume. Achieving homogeneous interaction of PANI with all @TiO_2_ and/or TiO_2_ nanoparticles through conventional mixing proves to be challenging due to TiO_2_ nanoparticles’ proclivity to agglomerate. Conversely, TEM microstructural imagery supports the consistent interaction between PANI and @TiO_2_ nanoparticles [63].

**Figure 11:**
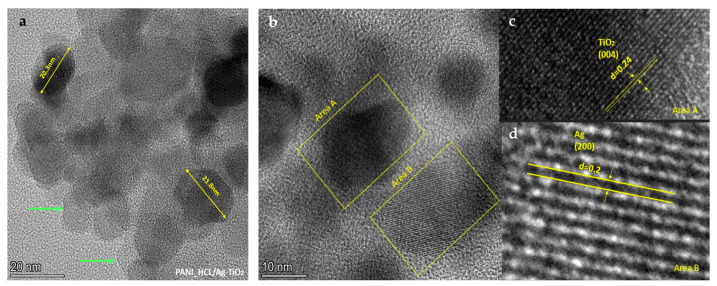
TEM images. (**a**) View of PANI_HCL/Ag-TiO_2_ particle, the green arrows indicate the formed polymeric coating, while with careful observation, the depth perspective (thickness) in this formation can be further appreciated. (**b**) View of the identified areas in the PANI_HCL/Ag-TiO_2_ sample, showing distinct lattice spacings of the crystalline phase of anatase in region A and silver in region B. (**c**,**d**) Magnified regions A and B.

TEM observations of PANI_HCL/Ag-TiO_2_ NP depict complex structures of amorphous and crystalline regions that include chemically modified TiO_2_ spherical particles. The chemically modified @TiO_2_ nanoparticles appear to be evenly dispersed within the PANI matrix, which prevents aggregation. A well-formed and defined particle with a diameter of ~20 nm is identified, consistent with the XRD findings (Figure 11a). The presence of particles of this size indicates successful particle synthesis on a nanoscale. The marked area A in Figure 11b shows the presence of the lattice plane (004), while in area B, the presence of silver crystals of the lattice plane (200) is observed. These findings align with XRD d-spacing results (Figure 11c,d and Table 8) obtained from TEM images align with the findings from XRD and micro-Raman analyses, also certifying the assembly’s structure [32,33,61,64,65,66]. The gray and white areas surrounding the spherical particles indicate the presence of polyaniline, which appears to encapsulate the crystalline particles. Especially, in Figure 11a, a polymeric structure (polyaniline) is indicated by green arrows (gray turning white at the boundaries), which can encapsulate inorganic nanoparticles [67,68,69,70,71,72].

**Figure 12:**
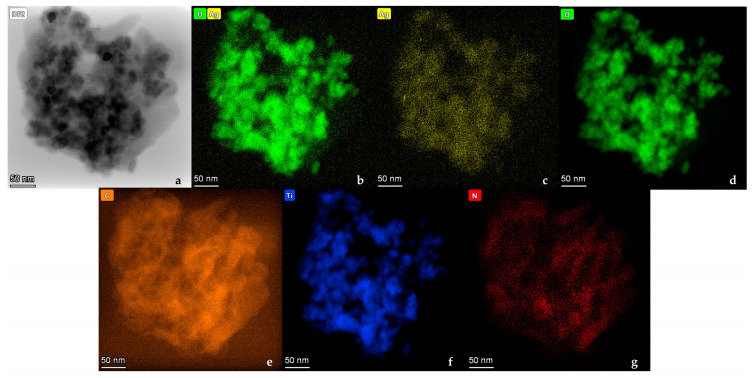
(**a**) TEM image; general view, (**b**–**g**) TEM element mapping.

**Figure 13:**
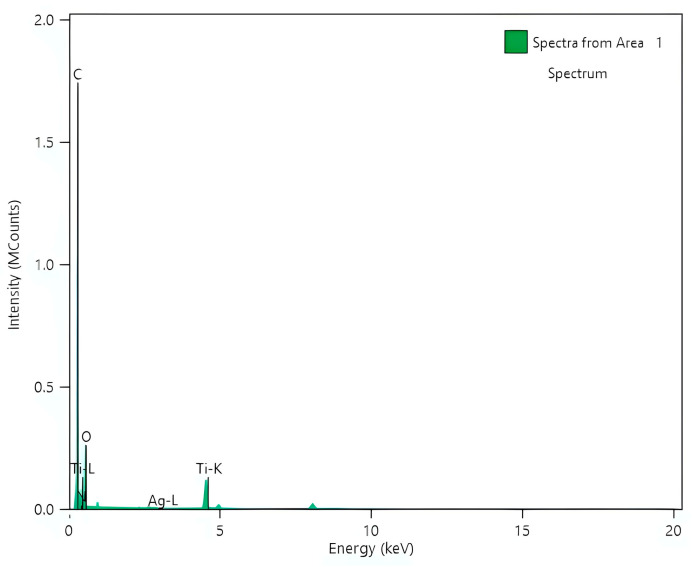
EDS analysis of PANI_HCL/AgTiO_2_.

Elemental mapping via EDS analysis (Figure 12a–g) unveiled the occurrence of C, N, O, Ag, as well as Ti, corroborating the effective production of composite photocatalysts. The existence of C and N on the nanostructured PANI_HCL/Ag-TiO_2_ surface signifies modification of the @TiO_2_ surface with polyaniline (C_6_H_8_N_2_). EDS analysis further affirmed the occurrence of C, N, Ti, as well as O (Figure 13), with a C-to-N ratio akin to that of polyaniline’s molecule (C: 79%, N: 16%, H: 5%) [73]. TiO_2_ content values in composite materials slightly fall short of the actual added amount due to the increase in PANI matrix’s weight during oxidative polymerization.

**Figure 14:**
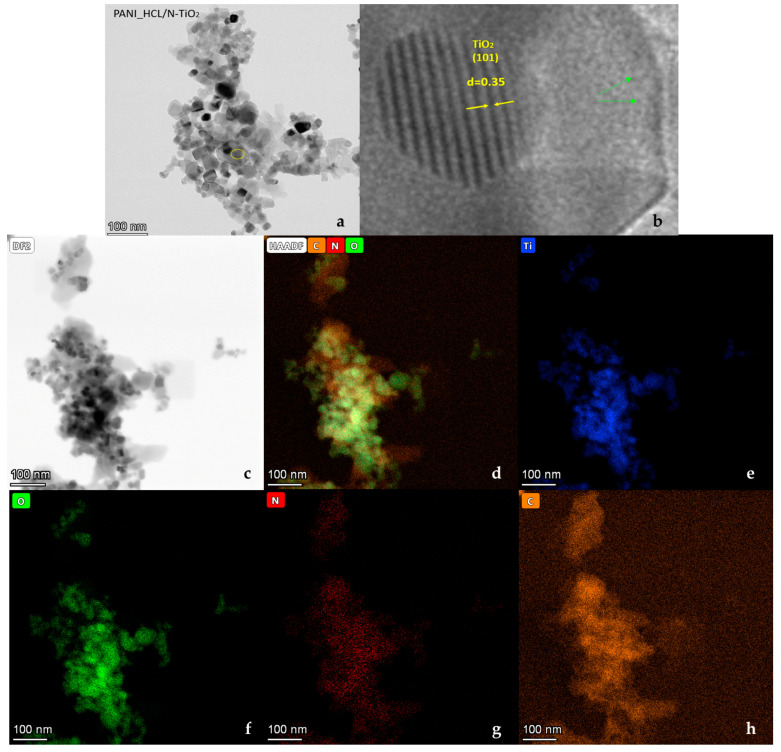
(**a**) TEM image; general view. (**b**) Magnification of the marked area of a delineated particle with visible crystallization at the (101) lattice plane of anatase. A lighter gray color indicates the presence of a polymer that appears to enclose the titanium dioxide nanoparticle. (**c**–**h**) TEM element mapping of PANI_HCL/N-TiO_2_.

**Figure 15:**
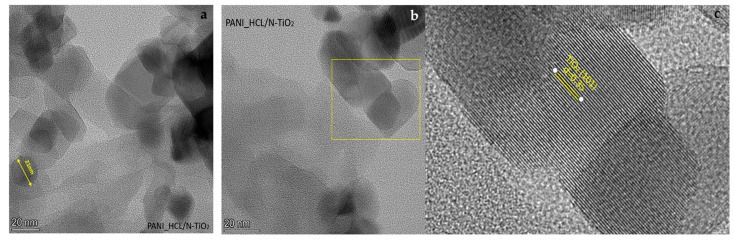
TEM images. (**a**) View of PANI_HCL/N-TiO_2_ particles. (**b**) Marked area showing the apparent presence of well-formed crystalline phase of anatase 001. (**c**) Magnification of the marked area.

**Figure 16:**
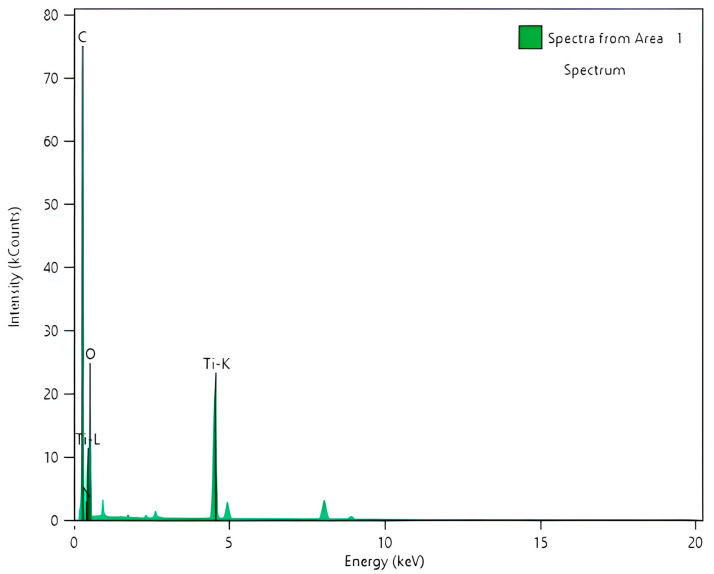
EDS analysis of PANI_HCL/N-TiO_2_.

In PANI_HCL/N-TiO_2_ composites (Figure 14a,b), encapsulation of N-TiO_2_ nanoparticles by PANI prevents aggregation, facilitated by repulsive forces between nanoparticles. Positively charged PANI-ES surface deposits on nano-N-TiO_2_ particles, inducing repulsion. Chemical modification of TiO_2_ can be inferred through XRD, FTIR, and micro-Raman spectra, discerning lattice distortions [16,61]. As it is shown in Figure 14a, a general view of the sample revealed well-formed and delineated particles, while magnification at a specific point unveiled the existence of the (101) crystal plane of anatase-TiO_2_. The lighter-colored area indicates the presence of a polymer enclosing the titanium dioxide nanoparticle. Also, in Figure 15a, another well-formed nanoparticle with a diameter of ~20 nm is observed, and the crystalline lattice corresponding to the 101 lattice of anatase is recognized in Figure 15b,c. The more careful morphological observation of the composite material by TEM reveals modestly doped TiO_2_ particles dispersed and possibly anchored in PANI’s chain. Specifically, Figure 14b indicates that the composite was fully synthesized since the distance between ≈0.35 nm in N-TiO_2_ corresponds to the (101) crystalline plane, while the blurry areas around revealed the covering membrane of polyaniline. The green arrows (Figure 14b) indicate the boundary of the formed polyaniline layer of a composite encapsulated particle. This is consistent with the EDS data, as areas with C and Ti are detected (Figure 16). Furthermore, TiO_2_ serves as a “template” for the homogenous aniline’s polymerization, and this morphology is favorable for increasing the active contact area for electrolyte ions, potentially provoking enhanced electrochemical activity. In the meantime, the outer layer of PANI might serve as a protective barrier for TiO_2_, preventing it from undergoing reducible dissolution, consequently leading to notable improvements in both electrochemical and photolytic robustness. Additionally, it can be observed that the composite particles may take relatively arbitrary shapes, in which case it is clear that there is repeatability due to the continuous surficial development of polyaniline’s polymeric chains on TiO_2_ particles, composing a continuous network. Therefore, the growth in the size of the enclosed nanoparticles is justifiable when compared to the size of the doped TiO_2_, with the total enlargement being influenced by the thickness of the externally formed layer, potentially linked to the PANI/TiO_2_ charge ratio [67,68,69,70,71,72].

**Figure 17:**
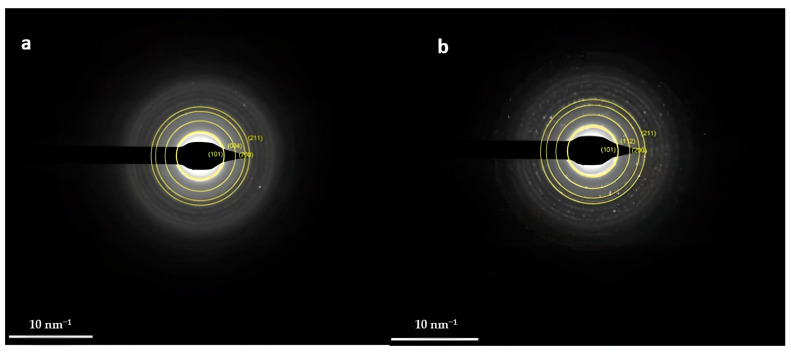
SAED patterns of (**a**) PANI_HCL/Ag-TiO_2_ (**b**) PANI_HCL/N-TiO_2_.

Selected area electron diffraction (SAED) patterns for both samples (Figure 17a,b) confirm the well-crystalline nature of TiO_2_ nanoparticles, also allowing d-spacing estimation.

**Table 8:** d-spacing values for PANI_HCL/Ag-TiO_2_ and PANI_HCL/N-TiO_2_ obtained from TEM image analysis of Figure 13.

Sample ID	d Spacing (Å)	hkl (Miller Indices)
PANI_HCL/Ag-TiO_2_	3.37	101
	2.38	004
	1.88	200
	1.67	211
PANI_HCL/N-TiO_2_	3.37	101
	2.35	112
	1.87	200
	1.67	211

### 3.2. Photocatalytic Activity Experiments

#### 3.2.1. RhB’s Photocatalytic Degradation

**Figure 18:**
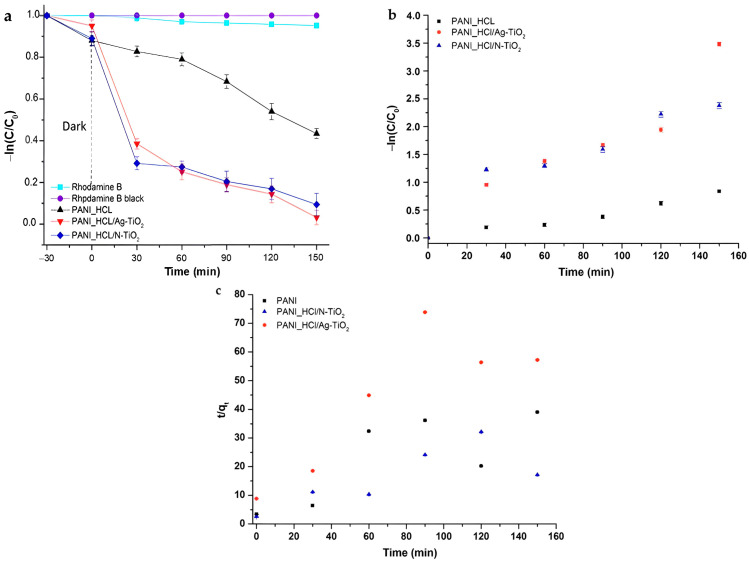
(**a**) Degradation curves of RhB for PANI_HCL, PANI_HCL/Ag-TiO_2_ and PANI_HCL/N-TiO_2_ as a function of time upon visible-light irradiation. Photolysis of RhB and its degradation under dark conditions were also examined. (**b**) Photocatalytic kinetic model for PANI_HCL, PANI_HCL/Ag-TiO_2_ and PANI_HCL/N-TiO_2_ based on (**b**) a pseudo-first-order model. (**c**) A pseudo-second-order model.

The photocatalytic trials were carried out at 25 °C and pH = 7.50 ± 0.01 [10,14,74] and the photocatalytic efficiency of the fabricated powders was examined upon visible-light illumination. The obtained data (Figure 18a) imply that the composite PANI_HCL/Ag-TiO_2_ exhibited enhanced photocatalytic degradation efficiency, achieving ≈ 97% elimination of RhB after 150 min.

The data illustrated in Figure 18a demonstrate that the PANI_HCL/Ag-TiO_2_ nanocomposites exhibited greater photoactivity compared to the samples containing nitrogen [33] and pure PANI_HCL. This observation could possibly be credited to the synergistic effect among PANI and nano-Ag–TiO_2_ particles. Nitrogen contributes to the generation of an e^−^-occupied intra-band gap that enables charge transfer among TiO_2_’s conduction (CB) and valence (VB) bands upon visible-light irradiation [16]. The existence of silver in the composite amplifies charge separation by scavenging e^−^ in TiO_2_’s CB, thus enhancing its photocatalytic attributes. The results demonstrate that the PANI_HCL/Ag-TiO_2_ photocatalyst exhibits the highest response towards RhB’s decolorization given the synergistic effect of photocatalysis and adsorption procedures [16,32,75,76,77]. Nevertheless, it is imperative to acknowledge that the PANI-HCL/N-TiO_2_ sample is also very effective, as the non-metal doping agent yields equally good results, presenting a slight reduction in efficiency (≈6%) and lower production costs.

The Langmuir–Hinshelwood equation, altered for reactions happening at the liquid-solid interface, is commonly used to represent pseudo-first kinetics [9,33] (Figure 18b) (Equation (4)):

(4) $$-ln\frac{C}{C_{0}}=k_{app}\times t$$

where C_0_ represents RhB’s initial concentration, C stands for RhB’s concentration at irradiation time t, and k_app_ corresponds to the apparent photo-induced degradation rate constant.

For colored compounds such as dyes, the rate of degradation typically increases until a critical concentration level is reached, beyond which it starts decreasing. This decrease can be ascribed to visible-light irradiation’s screening by RhB’s molecules prior to reaching the catalyst’s surface. However, modifying the catalyst’s concentration according to the organic compound’s concentration may lead to effective degradation, as the organic substance could be adequately adsorbed on the surface of the photocatalysts [78]. Most degradation studies use concentrations of organic compounds or pollutant dyes ranging from 10 to 200 mg/L that are comparable to pollutants’ concentrations typically detected in real sewage effluents. In this study, the concentration was set at 0.01 M. The calculated photo-induced degradation rate constant (k_app_), as well as the coefficient of the linear regression fitting (R^2^) for all particles are presented in Table 9. The linear kinetic model demonstrates a satisfactory fit across all experimental results, which is evident from the R^2^ values, while the PANI_HCL/Ag-TiO_2_ composite material presented the optimal photocatalytic efficiency. The removal efficiency was calculated applying Equation (5):

(5) $$Removal\;efficiency\left(\%\right)=\left(\frac{C_{0}-C}{C_{0}}\right)\times 100$$

The kinetics of photocatalytic experiments can also be attributed to the pseudo-second-order equation, outlined as follows (Equation (6)) [1]:

(6) $$\frac{t}{q_{t}}=\frac{1}{k_{2}q_{e}^{2}}+\frac{1}{q_{e}}t$$

where q_t_ (mg/g) and q_e_ (mg/g) represent the quantity of pollutant adsorbed at time t and at equilibrium respectively, while k_2_ denotes the rate constant (g/mg·min).

In comparison to the pseudo-first-order kinetics (Figure 18b), the R^2^ values derived from the pseudo-second-order kinetic analysis (Figure 18c) are notably lower. The kinetic parameters of the as-synthesized samples are outlined in Table 9. Based on the R^2^ values obtained from the kinetic investigations, the photocatalytic oxidation of RhB dye under visible-light irradiation for all examined samples conforms to pseudo-first-order reaction kinetics (Figure 18c).

**Figure 19:**
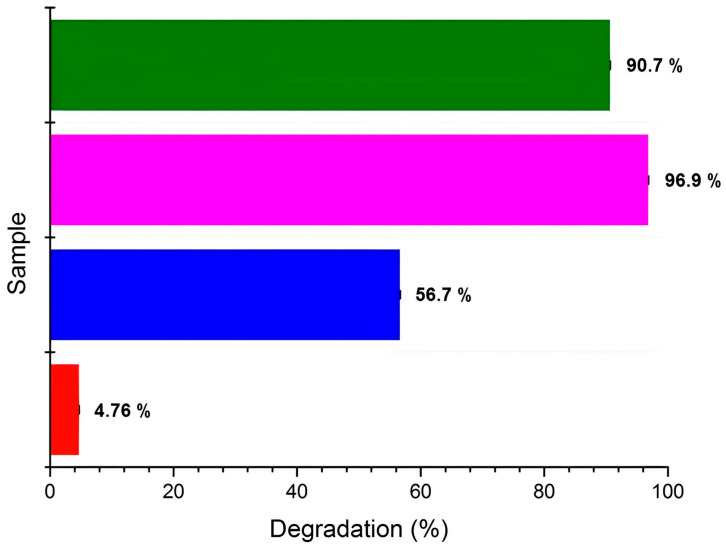
RhB’s degradation percentage after 150 min of visible-light irradiation, for PANI_HCL (in blue), PANI_HCL/Ag-TiO_2_ (in purple), and PANI_HCL/N-TiO_2_ (in green). RhB’s degradation percentage in the absence of the tested samples has been also included (in red).

The percentage of RhB degradation for each tested sample is depicted in Figure 19. According to the literature, the highly doping chemistry and the semi-crystalline phase of polyaniline exert a pivotal influence on the degradation mechanism of organic pollutants, owing to the absorbance capacity [79,80] and excellent efficiency, as our result reported. In the following table (Table 10), the photocatalytic efficiency of other reported nanocatalysts towards degradation of dyes under visible-light irradiation is shown, supporting the enhanced photocatalytic effectiveness of the as-synthesized samples tested within the present study.

#### 3.2.2. Reusability of PANI_HCL/@TiO_2_

**Figure 20:**
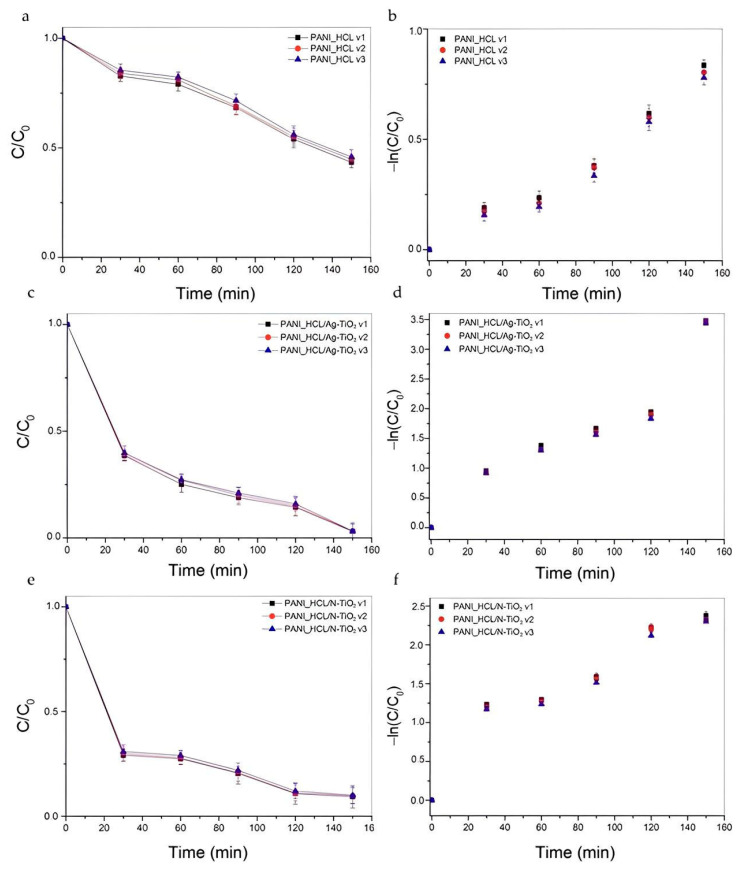
Reusability study. RhB’s degradation curves for (**a**) PANI_HCL, (**c**) PANI_HCL/Ag-TiO_2_ and (**e**) PANI_HCL/N-TiO_2_ as a function of time upon visible-light irradiation after three subsequent runs. Photocatalytic kinetic model study for (**b**) PANI_HCL, (**d**) PANI_HCL/Ag-TiO_2_, and (**f**) PANI_HCL/N-TiO_2_ based on a linear pseudo-first-order model.

The economic viability of the photocatalytic process is remarkably influenced by both photostability and the longevity of the catalyst, which are essential factors to consider [63,82,83]. Reusability tests were also performed under visible-light irradiation conditions, utilizing a catalyst loading of 5 mg, maintaining a pH of 7.50 ± 0.01, and an initial RhB concentration of 0.01 M. Following each experimental trial, the examined catalysts underwent vacuum filtration to collect them, pursued by rinsing with distilled water and subsequent drying at 60 °C. Additionally, catalysts’ weight loss was estimated and found to be approximately 3%. The process was repeated thrice (three runs), and the obtained results are depicted in Figure 20a,c,e. The obtained k_app_ and R^2^ values are presented in Table 11, relying on the linear pseudo-first-order kinetic model that was previously analyzed (Figure 20b,d,f). The data indicated that the studied PANI/@TiO_2_ photocatalysts did not present a noticeable reduction in their performance during RhB’s degradation, proving their robustness.

#### 3.2.3. Proposed Photocatalytic Mechanism

As previous studies have indicated, the process of photocatalytic degradation is quite complex. In some cases, the photocatalytic mechanism for certain catalysts is still not fully understood. In order to investigate the potential photocatalytic mechanisms for RhB under visible light for the produced composite materials, we conducted a series of photocatalytic tests. Reactive oxygen species (ROS), such as hydroxyl (•OH) and superoxide (•O_2_^−^) radicals, as well as holes (h^+^), comprise resilient oxidizing agents leading to the effective degradation of various organic pollutants [84].

**Figure 21:**
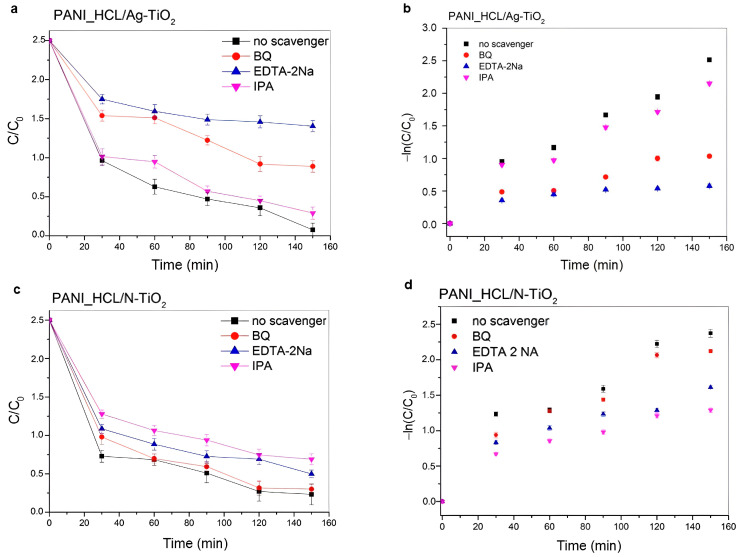
RhB’s degradation curves in the presence of (**a**) PANI_HCL/Ag-TiO_2_ and (**c**) PANI_HCL/N-TiO_2_ and ROS scavengers (EDTA-2Na, BQ and IPA). Photocatalytic kinetic study of (**b**) PANI_HCL/Ag-TiO_2_ and (**d**) PANI_HCL/N-TiO_2_ based on a linear pseudo-first-order model.

In this study, different scavengers (Table 12) were used to certify the involved ROS towards RhB’s degradation upon visible-light irradiation, when PANI_HCL/Ag-TiO_2_ and PANI_HCL/N-TiO_2_ samples were present (Figure 21).

**Figure 22:**
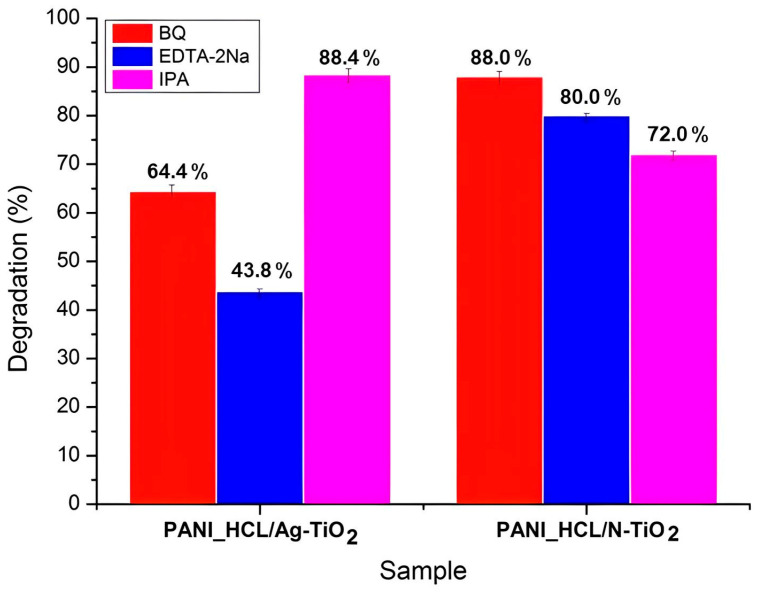
RhB’s degradation percentage utilizing PANI_HCL/Ag-TiO_2_ and PANI_HCL/N-TiO_2_ samples in the presence of EDTA-2Na (blue), BQ (red) and IPA (purple) scavengers.

**Figure 23:**
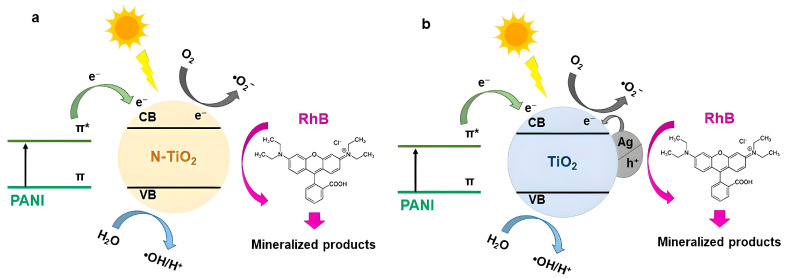
Proposed photocatalytic mechanism of (**a**) PANI_HCL/Ν-TiO_2_ and (**b**) PANI_HCL/Ag-TiO_2_ under visible-light irradiation for the degradation of RhB.

According to Figure 21, RhB’s degradation was decreased upon the addition of BQ, IPA, and EDTA-2Na into the reaction solution at a RhB-scavenger concentration equal to 0.001 M, as similarly reported in previous studies [17,46,85,86]. Notably, in the case of PANI_HCL/Ag-TiO_2_ sample, RhB’s degradation effectiveness was reduced when EDTA-2Na was poured into the reaction solution, suggesting that h^+^ constitute the dominant ROS involved in the degradation process (Figure 21a). Similarly, for PANI_HCL/N-TiO_2_, RhB’s degradation decreased after adding an IPA scavenger (Figure 21c). The proposed mechanism of RhB’s photodegradation is based on both the experimental observations of photocatalytic degradation and the scavenging tests, suggesting that the enhanced photocatalytic activity is mainly attributed to PANI’s doping [76]. The k_app_ and R^2^ values for PANI_HCL/Ag-TiO_2_ and PANI_HCL/N-TiO_2_ are presented in Table 13, in line with the linear pseudo-first-order kinetic model (Figure 21b,d). Table 14 summarizes the ROS that were generated in the presence of the two as-mentioned samples. RhB’s degradation percentage for each sample and for the three tested scavengers is shown in Figure 22. Figure 23 depicts the proposed photocatalytic mechanism of the degradation of RhB in the presence of PANI_HCL/Ν-TiO_2_ and PANI_HCL/Ag-TiO_2_ under visible-light irradiation.

### 3.3. Biocombatibility Test

#### Effect on Cytotoxicity

For investigating nanocomposites’ biocompatibility, an MTT colorimetric assay was applied on HEK293 and FF95 normal cells in the presence of increasing concentrations of PANI_HCL, PANI_HCL/Ag-TiO_2_, and PANI_HCL/N-TiO_2_ before and after of visible-light irradiation. Thus, 10,000 cells for each sample were cultured. Then, the cells were subjected to ascending concentrations of the as-developed materials (PANI_HCL, PANI_HCL/Ag-TiO_2_, and PANI_HCL/N-TiO_2_) within the 0–0.75 mg/mL range. Visible-light irradiation for a duration of 30 min allowed the photo-activation of the materials in the appropriate samples. Based on the widely used protocol [87], cell viability (%) was evaluated as a fraction of the optical density that was measured for any sample of treated cells compared to that of the untreated ones.

**Figure 24:**
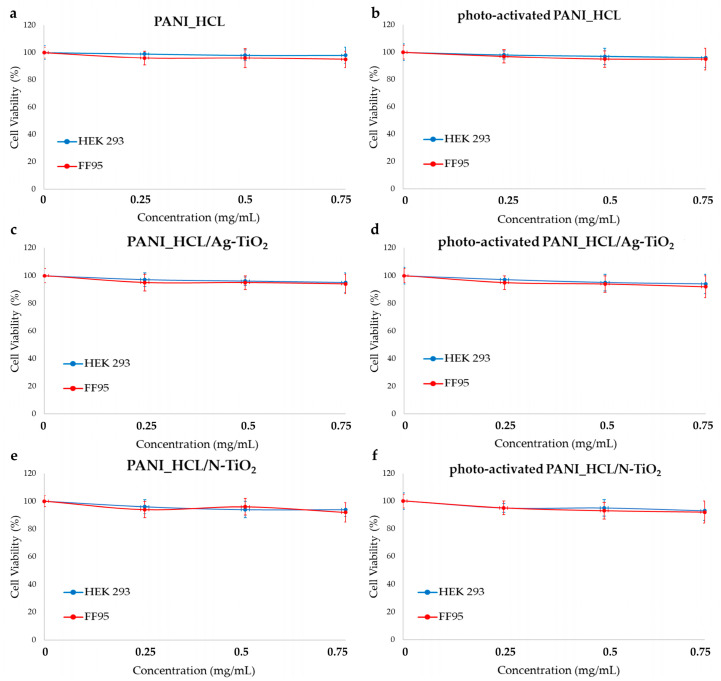
Effect of (**a**,**b**) PANI_HCL, (**c**,**d**) PANI_HCL/Ag-TiO_2_, and (**e**,**f**) PANI_HCL/N-TiO_2_ on cell viability. MTT colorimetric assay was utilized to resolve cell viability’s percentage of HEK293 and FF95 cells in the presence of nanocomposites’ escalating concentrations before (**a**,**c**,**e**) and after (**b**,**d**,**f**) photoactivation with visible light. No significant effect on cell viability was observed.

HEK 293 and FF95 cells were unaltered in the aforementioned range of concentrations when PANI_HCL (Figure 24a) or photoactivated PANI_HCL (Figure 24b), PANI_HCL/Ag-TiO_2_ (Figure 24c) and photo-activated PANI_HCL/Ag-TiO_2_ (Figure 24d), PANI_HCL/N-TiO_2_ (Figure 24e), and photo-activated PANI_HCL/N-TiO_2_ (Figure 24f) were present. The series of experiments showed high repeatability, indicating that these materials seem to be non-toxic to human skin and kidneys. According to other studies, PANI was not expected to be toxic in HEK 293 since cells treated with PANI were not affected either in morphology or in their proliferation rate [88]. PANI is considered biocompatible since it has been proven to be non-toxic when tested on the mouse embryonic fibroblast cell line (NIH/3T3) and the embryonic stem cell ES R1 line (ESc) [89]. According to our previous study, Ag-TiO_2_ nanoparticles did not also affect HEK 293 cells [32]. Hence, the composite material PANI_HCL/Ag-TiO_2_ was non-toxic to HEK 293 cells. N-TiO_2_ nanoparticles are also non-toxic on HEK293 as various studies suggest [90]. Therefore, it is very promising that these innovative composites (PANI_HCL/Ag-TiO_2_ and PANI_HCL/N-TiO_2_) are also non-cytotoxic since they can be utilized in several environmentally friendly applications, such as wastewater treatment photocatalytic pigments, as well as in biomedical applications exploiting their high photocatalytic performance.

## 4. Conclusions

PANI-encapsulated @TiO_2_ composite particles were efficiently synthesized through chemical oxidation polymerization and characterized applying XRD, micro-Raman, FT-IR, DRS, DLS, and TEM techniques. Thus, their morphology and physicochemical characteristics were studied to also confirm the successful fabrication of the composite materials. Based on the XRD analysis, pure PANI_HCL presents a semi-crystal structure. The average crystallite size of chemically modified titania particles in PANI_HCL/Ag-TiO_2_ and PANI_HCL/N-TiO_2_ composites was estimated at ≈19.4 and ≈20.38 nm, respectively. Through micro-Raman studies using the excitation line of 633 nm, the characteristic bands of the TiO_2_ phase were detected in both types of composites, PANI_HCL/Ag-TiO_2_ and PANI_HCL/N-TiO_2_ samples, and were also overlapped by those bands that are related to polyaniline, showing the co-existence of both phases in the composites. These results were further confirmed by FT-IR data. The energy band gaps of PANI_HCL/Ag-TiO_2_ and PANI_HCL/N-TiO_2_ were 2.02 and 2.14 eV, allowing visible-light photo-activation. The zeta potential values of the samples (PANI_HCL: −26.1 ± 0.9 mV, PANI_HCL/Ag-TiO_2_: −29.9 ± 0.7 mV, PANI_HCL/N-TiO_2_: −37.8 ± 1.2 mV) showed that the samples were significantly stable. TEM images revealed the homogeneous interaction of PANI with the entire doped TiO_2_. EDS analysis validated the occurrence of C, N, O, Ag, and Ti, validating the effective synthesis of crystalline photocatalytic nanoparticles encapsulated in a polyaniline matrix. The polyaniline layer does not seem to affect the morphology or the crystal structure of the inorganic particles but aids in the retention of doped TiO_2_ nanoparticles. The size of the composite encapsulated particles appears larger than that of the non-encapsulated ones, which is expected due to the development of polyaniline chains on their surface, eventually forming a layer capable of encapsulating the inorganic particles.

RhB’s photocatalytic degradation was investigated upon visible-light activation. When PANI_HCL/Ag-TiO_2_ and PANI_HCL/N-TiO_2_ samples were present, the pollutant was degraded by ≈97% and 91%, respectively, after 150 min of irradiation. The same experiment was repeated in the presence of ROS scavengers, proving that •OH was the prevailing ROS in the case of PANI_HCL/Ag-TiO_2_ and the positive holes for PANI_HCL/N-TiO_2_. Furthermore, the reusability of photocatalysts was studied after three runs with very promising results. In particular, PANI-encapsulated @TiO_2_ nanocomposites are capable of being reused for a minimum of three subsequent rounds, without considerable efficiency loss. For an eco-friendly, cost-effective application, this feature is very important.

Finally, for examining the composites’ biocompatibility, two normal cell lines were used (HEK293 and FF95). There was not any cytotoxic effect, when PANI, PANI_HCL/Ag-TiO_2_, and PANI_HCL/N-TiO_2_ samples were present. Thus, these materials are not harmful to cells, so they could be considered biocompatible. This result allows the further investigation of the possible use of the developed materials in environmental (wastewater treatment, photocatalytic pigments, etc.) and biomedical applications (antibacterial applications, etc.), avoiding any harmful effects in health tissues, in the environment, and in any living organism. Overall, from the observations of this study, we could say that the composite particles exhibit enhanced photocatalytic activity due to their unique structures and compositions, enabling efficient degradation of organic pollutants and the conversion of toxic compounds into harmless substances. The energy band gaps of the composites) enable visible-light photoactivation, expanding the application of photocatalysis to a wider range of environmental conditions. The composites demonstrate promising reusability for at least three subsequent rounds without significant efficiency loss, making them cost-effective and eco-friendly. Additionally, they are biocompatible, indicating their potential use in various applications without harming cells or the environment.

The development of PANI-encapsulated chemically modified TiO_2_ composite particles represents a significant innovation in the field of photocatalysis. These materials exhibit enhanced photocatalytic activity, visible-light photoactivation, and reusability, making them suitable for various environmental and biomedical applications. Their biocompatibility further highlights their potential for safe and effective use in various applications, avoiding any harmful effects on health tissues, the environment, and living organisms.

## Figures and Tables

**Table 1:** Calculated parameters for PANI_HCL, PANI_HCL/Ag-TiO_2_ and PANI_HCL/N-TiO_2_ samples, obtained through the XRD analysis.

Sample ID	Average Crystallite Size (nm)	FWHM *	Crystallinity (%)
PANI_HCL	-	-	25.30
PANI_HCL/Ag-TiO_2_	19.4 ± 5.3 × 10^−1^	0.7198	73.44
PANI_HCL/N-TiO_2_	20.38 ± 4.4 × 10^−1^	0.5355	43.33

* Average value of total calculated values for several well distinct peaks.

**Table 2:** Main bands in the Raman spectrum and their assignment for the tested samples (PANI_HCL, PANI_HCL/Ag-TiO_2_ and PANI_HCL/N-TiO_2_).

Wavenumbers (cm^−1^)	Assignment
148	E_g(1)_ anatase-TiO_2_ crystal phase
580	Ring deformation
1164	C–H distortion vibration of a quinone ring in emeraldine
1585	C–C ring stretching vibrations
1620	C–C stretching in B

**Table 3:** Main bands in the FT-IR spectrum and their assignment for the tested samples (PANI_HCL, PANI_HCL/Ag-TiO_2_ and PANI_HCL/N-TiO_2_).

Wavenumbers (cm^−1^)	Shifted Wavenumbers in the Composites (cm^−1^)	Assignment
1558	1562	C=C (Q)
1469	1472	C=C (B)
1281	1294	C–H in plane of deformation
1234		C–N vibrations in BBB
1129	1179	in plane C–H bending
777	795	vibrations of C–H bonds
484		Aromatic ring’s C–N–C bonding mode

**Table 4:** E_g_ values of the as-synthesized samples.

Sample ID	E_g_ (eV)
PANI_HCL	2.74
PANI_HCL/Ag-TiO_2_	2.02
PANI_HCL/N-TiO_2_	2.14

**Table 5:** Zeta potential values for PANI_HCL, PANI_HCL/Ag-TiO_2_, and PANI_HCL/N-TiO_2_ samples.

Sample ID	Zeta Potential (mV)—RT
PANI_HCL	−26.1 ± 0.9
PANI_HCL/Ag-TiO_2_	−29.9 ± 0.7
PANI_HCL/N-TiO_2_	−37.8 ± 1.2

**Table 6:** Percentage of carbon and nitrogen components concentration derived from C1s and N1s XPS spectra deconvolution.

Sample ID	Carbon Components				Nitrogen Components	
	C–C	C–N	C=N	C=O	Pyrolic nitrogen	C–N–C and N-TiO_2_
PANI_HCL/N-TiO_2_	45.1	30.7	15.5	9.0	21.0	79.0
PANI_HCL/Ag-TiO_2_	46.2	32.0	15.6	7.0	19.7	80.3

**Table 7:** The % atomic concentration of carbon, oxygen, nitrogen, silver and titanium.

Sample ID	C	O	N	Ag	Ti
Ag-TiO_2_	27.4	46.8	-	8.7	17.2
PANI_HCL/N-TiO_2_	63.5	23	6	-	7.5
PANI_HCL/Ag-TiO_2_	56.5	24.7	-	6.2	8.1

**Table 9:** Calculated kinetic parameters for all synthesized nanocomposites.

Sample ID	Pseudo-First-Order Kinetic Model		Pseudo-Second-Order Kinetic Model	
	R^2^	k_app_ (min^−1^)	R^2^	k_2_ (g/mg·min)
PANI_HCL	0.96	5.34 × 10^−3^	0.50	2.13 × 10^−1^
PANI_HCL/Ag-TiO_2_	0.90	1.96 × 10^−2^	0.60	3.66 × 10^−1^
PANI_HCL/N-TiO_2_	0.87	1.44 × 10^−2^	0.46	1.43 × 10^−1^

**Table 10:** Photocatalytic effectiveness of reported nanocatalysts upon visible-light irradiation.

Nanocatalyst	Remarks	Reference
N-TiO_2_ NPs	▪ Highest degradation percentage: 60% ▪ Tested pollutant: RhB ▪ Duration of photocatalytic trial: 150 min	[36]
N-TiO_2_ NPs	▪ Highest degradation percentage: 74.76% ▪ Tested pollutant: RhB ▪ Duration of photocatalytic trial: 240 min	[1]
N,S-TiO_2_ NPs	▪ Highest degradation percentage: 92.83% ▪ Tested pollutant: RhB ▪ Duration of photocatalytic trial: 240 min	[1]
TiO_2_/SiO_2_ NPs	▪ Highest degradation percentage: 100% ▪ Tested pollutant: RhB ▪ Duration of photocatalytic trial: 210 min	[5]
Ag-TiO_2_/microgel	▪ Highest degradation percentage: 95% ▪ Tested pollutant: RhB ▪ Duration of photocatalytic trial: 150 min	[30]
ZnO NPs	▪ Highest degradation percentage: 100% ▪ Tested pollutant: RhB ▪ Duration of photocatalytic trial: 210 min	[2]
Fe_2_O_3_/TiO_2_/clinoptilolite	▪ Highest degradation percentage: 92.9% ▪ Tested pollutant: Acid Black 172 ▪ Duration of photocatalytic trial: 120 min	[81]
PANI_HCL/Ag-TiO_2_	▪ Highest degradation percentage: 96.9% ▪ Tested pollutant: RhB ▪ Duration of photocatalytic trial: 150 min	Present study
PANI_HCL/N-TiO_2_	▪ Highest degradation percentage: 90.7% ▪ Tested pollutant: RhB ▪ Duration of photocatalytic trial: 150 min	Present study

**Table 11:** Calculated k_app_ and R^2^ values obtained after the reusability study for all produced nanocomposites.

Sample ID	R^2^	k_app_ (min^−1^)
Run 1		
PANI_HCL	0.96	5.34 × 10^−3^
PANI_HCL/Ag-TiO_2_	0.90	1.96 × 10^−2^
PANI_HCL/N-TiO_2_	0.87	1.44 × 10^−2^
Run 2		
PANI_HCL	0.96	5.19 × 10^−3^
PANI_HCL/Ag-TiO_2_	0.89	1.95 × 10^−2^
PANI_HCL/N-TiO_2_	0.85	1.30 × 10^−2^
Run 3		
PANI_HCL	0.95	5.10 × 10^−3^
PANI_HCL/Ag-TiO_2_	0.89	1.93 × 10^−2^
PANI_HCL/N-TiO_2_	0.85	1.29 × 10^−2^

**Table 12:** Utilized scavengers.

Scavenger	Targeted ROS
1,4-benzoquinone (BQ)	●O_2_^−^
Isopropyl alcohol (IPA)	●OH
Ethylenediaminetetraacetic acid disodium salt (EDTA-2Na)	h^+^

**Table 13:** Calculated k_app_ and R^2^ values of PANI_HCL/Ag-TiO_2_ and PANI_HCL/N-TiO_2_ composites for all tested scavengers.

Sample ID	Scavenger	R^2^	k_app_ (min^−1^)
PANI_HCL/Ag-TiO_2_	BQ	0.90	6.59×10^−3^
	EDTA-2Na	0.70	3.33×10^−3^
	IPA	0.70	3.30×10^−3^
PANI_HCL/N-TiO_2_	BQ	0.90	1.34×10^−2^
	EDTA-2Na	0.83	9.17×10^−3^
	IPA	0.85	7.80×10^−3^

**Table 14:** ROS generated in the presence of PANI_HCL/Ag-TiO_2_ and PANI_HCL/N-TiO_2_, appearing in descending order of prevalence.

Sample ID	ROS
PANI_HCL/Ag-TiO_2_	h^+^ > ●O_2_^−^ > ●OH
PANI_HCL/N-TiO_2_	●OH > h^+^ > ●O_2_^−^

**Figure A2:**
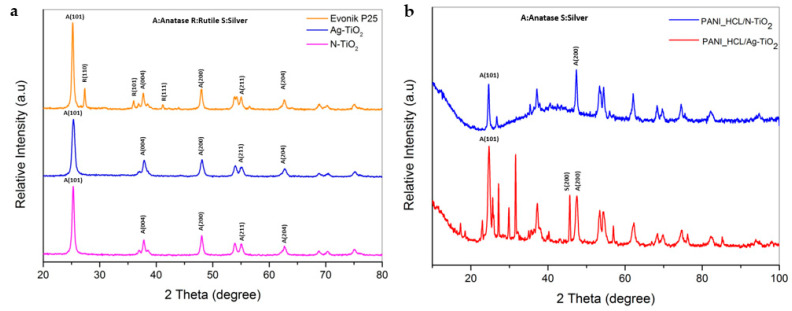
(**a**) XRD diffractograms of Evonik P25 (in orange), Ag-TiO_2_ (in blue) and N-TiO_2_ (in pink) [32,33], (**b**) XRD diffractograms of PANI_HCL/Ag-TiO_2_ (in red), and PANI_HCL/N-TiO_2_ (in blue) after completing the photocatalytic tests.

## Data Availability

Data is contained within the article.

## Appendix A

The analysis and comparison of samples of chemically modified TiO_2_ with Ag or N in relation to the commercial titanium dioxide Evonik P25 structure (75% anatase and 25% rutile) show shifts in the main band of the anatase (Figure A1a). Comparative to the spectra of the composite samples at the 532 line, an additional shift is observed in their main band of the anatase at a higher wavenumber (148 cm^−1^), possibly due to the sensitization of the PANI_HCL in the TiO_2_. The analysis of Ag-doped and N-doped TiO_2_ samples was not possible with a laser at 633 nm due to intense fluorescence.

Regarding the examination of photocatalysts after completing the photocatalytic tests for reusability potential, the XRD pattern remains unaltered (Figure A2b). This indicates the stability of the photocatalysts during their application in photocatalytic processes for the degradation of RhB aqueous solution under visible-light irradiation.
